# Gene Network Rewiring to Study Melanoma Stage Progression and Elements Essential for Driving Melanoma

**DOI:** 10.1371/journal.pone.0142443

**Published:** 2015-11-11

**Authors:** Abhinav Kaushik, Yashuma Bhatia, Shakir Ali, Dinesh Gupta

**Affiliations:** 1 Bioinformatics Laboratory, Structural and Computational Biology Group, International Centre for Genetic Engineering and Biotechnology, New Delhi, 110067, India; 2 Department of Biochemistry, Faculty of Science, Jamia Hamdard, New Delhi, 110062, India; Universidade de São Paulo, BRAZIL

## Abstract

Metastatic melanoma patients have a poor prognosis, mainly attributable to the underlying heterogeneity in melanoma driver genes and altered gene expression profiles. These characteristics of melanoma also make the development of drugs and identification of novel drug targets for metastatic melanoma a daunting task. Systems biology offers an alternative approach to re-explore the genes or gene sets that display dysregulated behaviour without being differentially expressed. In this study, we have performed systems biology studies to enhance our knowledge about the conserved property of disease genes or gene sets among mutually exclusive datasets representing melanoma progression. We meta-analysed 642 microarray samples to generate melanoma reconstructed networks representing four different stages of melanoma progression to extract genes with altered molecular circuitry wiring as compared to a normal cellular state. Intriguingly, a majority of the melanoma network-rewired genes are not differentially expressed and the disease genes involved in melanoma progression consistently modulate its activity by rewiring network connections. We found that the shortlisted disease genes in the study show strong and abnormal network connectivity, which enhances with the disease progression. Moreover, the deviated network properties of the disease gene sets allow ranking/prioritization of different enriched, dysregulated and conserved pathway terms in metastatic melanoma, in agreement with previous findings. Our analysis also reveals presence of distinct network hubs in different stages of metastasizing tumor for the same set of pathways in the statistically conserved gene sets. The study results are also presented as a freely available database at http://bioinfo.icgeb.res.in/m3db/. The web-based database resource consists of results from the analysis presented here, integrated with cytoscape web and user-friendly tools for visualization, retrieval and further analysis.

## Introduction

Advance malignant melanoma represents a deadly cancer state due to its high dissemination potential and increasing therapy resistance [1]. In general, melanoma initiates with marked disruption of cellular homeostatic mechanisms leading to a malignant transformation of skin melanocytes (cutaneous non-metastatic; CnM). Melanoma initiation is followed by its proliferation to different layers of skin via multiple growth phases (cutaneous metastasis; CM) and finally to lymph nodes (LN), which metastasize tumour to different organs [2].

After the advent of the ‘*omics*’ era, despite several attempts to identify gene signatures and genetic changes in melanoma, inconsistencies in the results still exists [3–5]. The likely reason for the inconsistency is the diverse histological types and multivariate nature of melanoma, making consensus view about melanoma gene expression behaviour, a challenge, which also hinders development of novel anti-melanoma therapies.

Historically, high-throughput (HT) melanoma studies have been mainly confined to identification and validation of mutated genes and multitude of Differentially Expressed (DE) genes for various classified melanomas or melanoma sub-types. Although the information about DE genes enhance our understanding of diseases, recent studies indicate that alteration in gene activity is not limited to its fold change in expression alone and the interactomes undergo re-wiring as a result of cellular or adaptive response [6]. Systems biology offers a range of alternative methods that allows identification of such disease linked genes characterised by abnormal linked behaviour. The new systems biology methods based on gene co-expression values allows prediction of gene essentiality beyond modules and hubs; which even helps generate novel hypotheses for disease mechanisms [7].

**Fig 1:**
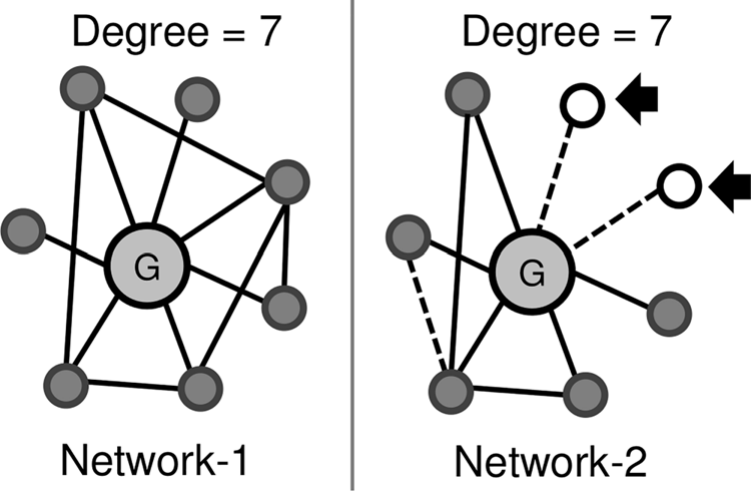
Gene connections in biological networks are dynamic and may show altered co-expression under variable conditions, even if genes shows no significant differential co-expression. Gene connections are marked as lines, new genes marked as hollow circles (marked with arrows) and dotted lines represent new connections.

It has been found that in a normal cellular homeostasis, the genes display a robust set of correlated oscillations, herein referred as network connections, which are disrupted in diseases [7, 8]. Thus, dysregulation linked with disease states cause differential correlation of various genes due to their altered gene expression pattern leading to reformation of network wiring circuitry [9]. It is expected that the genes that amplify its connectivity or expression in disease as compared to that in control stages are more likely to be involved in disease progression. In such stress conditions, several genes lose connections in the perturbed network while some may even gain alternate sets of connections [10]. Fig 1 illustrates the differential connectivity in network-1 as compared to network-2, which shows that some connections are lost while new connections (even new nodes) may be established in the altered network-2. Additional experimental evidence for this concept is a report by Hudson *et al*. [11], who conducted differential wiring analysis of expression data to correctly predict myostatin as the gene containing the causal mutation, characterised by differential wiring rather than differential expression. Anglani *et al*. described the loss of gene connections as a measure of its molecular dysfunction in five different cancers, which occurs when genes lose its role as an effector [12]. The study highlighted that oncogenic alterations in coding regions and post-translational modifications can modify gene connections and hence functions, without any changes in expression levels. In another study, Kim *et al*. correlated the gene essentiality with rewiring in yeast and mouse interactome and suggested that circuitry rewiring allow genes to integrate into alternative pathways [13]. A similar study utilizes the number of connections gained and lost by a gene to measure its overall rewiring score [8]. The analysis revealed that the disease genes tend to rewire its network circuit and hence gene-rewiring scores are suitable for gene prioritization. Moreover, previously it was found that ERK-MAPK activity in melanoma leads to pathway rewiring which results in its connection of ERK to JNK pathways- this observation lead to identification of additional melanoma therapeutic targets [14]. Thus, the available literature suggests that not only genes but the associated pathways too get dysregulated after connection rewiring, understanding of which is a critical step in designing novel and more effective therapeutics [15]. An additional advantage of exploiting the network based system biology approach for the prediction of genes involved in disease is that the sensitivity of reconstructed network increases with heterogeneity in expression dataset, which is a common feature of melanoma samples [5, 16].

There is no report of any study, which attempts to identify and measure the network dependent dynamicity and reproducibility of such potential and novel disease genes or gene sets (i.e. pathways) involved in melanoma progression, based on the analysis of heterogeneous melanoma datasets. In this study we exploited the publicly available cross-platform melanoma transcriptomic data (sample size > 600, grouped into four different stages *per se* melanoma progression), to decipher differentially wired genes in each stage.

In this study we have identified gene sets that are rewired and connected similarly in mutually exclusive datasets. Moreover, we have investigated the differential network properties of functional clusters developed from the shortlisted disease genes. The deviated network profile analysis assisted in identification of important stage specific pathway terms involved in stage transition. We demonstrate that there is a sudden increase in quantitative complexity of the predicted pathways with tumour progression especially after the onset of metastasis. Such prioritized/ranked gene clusters were found to possess strong disease association on the basis of disease linkage analysis. We also attempted to understand the dynamicity and flow of ranked pathway terms enriched during melanoma progression. The complete results are presented as a SQL database using DHTML and cytoscape-web [17] for user friendly visualization, retrieval and further analysis. The database resource is freely available at http://bioinfo.icgeb.res.in/m3db/.

## Results

Melanoma gene expression data collected from publicly available data repositories was manually stratified into four different stages, in the order of melanoma progression involving normal skin melanocytes (N) and the three transition events {*N* → *CnM* → *CM* → *LN*}. Table 1 shows the number of cross-platform gene expression dataset sizes and series, representing each stage and different sources (for details of each of the study data set, see S1 File). We performed estimation and removal of batch effect resulting from different experimental conditions, prior to the data analysis. The results are summarized in S1 Fig, which suggests that a large proportion of genes were affected with batch effect, removed by ComBat batch adjustment (see materials and methods).

**Table 1:** Dataset description.

Melanoma stage	Dataset size	Series	Platforms^†^
Normal	97	13	07^[^ ^*a*^ ^,^ ^*b*^ ^,^ ^*c*^ ^,^ ^*d*^ ^,^ ^*e*^ ^,^ ^*f*^ ^,^ ^*g*^ ^]^
Cutaneous non-Metastatic	183	09	05^[^ ^*d*^ ^,^ ^*e*^ ^,^ ^*f*^ ^,^ ^*h*^ ^,^ ^*g*^ ^]^
Cutaneous Metastatic	246	10	05^[^ ^*d*^ ^,^ ^*e*^ ^,^ ^*f*^ ^,^ ^*h*^ ^,^ ^*g*^ ^]^
Lymph Node Metastatic	116	07	05^[^ ^*a*^ ^,^ ^*c*^ ^,^ ^*d*^ ^,^ ^*e*^ ^,^ ^*h*^ ^,^ ^*i*^ ^]^

† Microarray platforms used by submitter.

***a***:GPL10558

***b***:GPL4133

***c***:GPL5175

***d***:GPL570

***e***:GPL571

***f***:GPL6883

***g***:GPL96

***h***:GPL1708

***i***:GPL6884

### Meta-analysis revealed a novel DE melanoma gene signature

**Fig 2:**
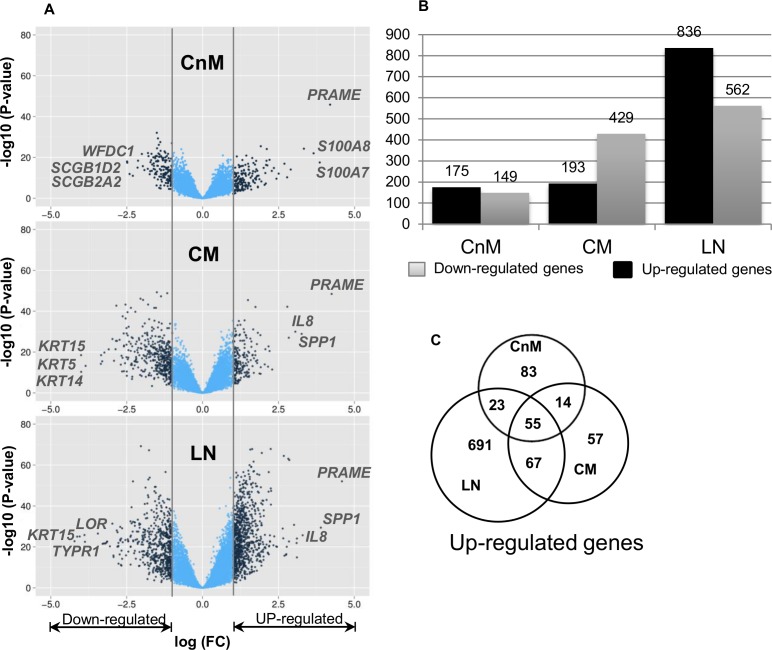
A. Scatter plot of log2 ratio (fold change; FC) versus adjusted p-value to measure the associated significant differential expression of each gene. Genes which are significantly differentially expressed (DE; log(FC) > 1 or < 1; adj.p value < 0.05) are shown in dark blue color B. Significantly DE gene count (up- and down-regulated) in each stage. C. Number of up-regulated genes conserved across different melanoma stages.

We initiated the analysis by checking the dataset quality and validation by prediction of known melanoma markers amongst statistically significant DE genes (logFC > 1 and adj.p < 0.05) between control (N) and different stages of melanoma (CnM, CM and LN). Comparative analysis highlights several stage specific changes (Fig 2). We identified 324, 622 and 1398 DE genes in CnM, CM and LN, respectively. We observed that despite the low fold change threshold, only 55 genes are expressed across all the stages of melanoma and indicate that the genes are essentially required for melanoma progression.

We looked for melanoma biomarkers and known cancer related genes in the conserved gene set (n = 55) and found several unique sets of genes previously reported to be melanoma or cancer linked. The gene set is enriched with several melanoma-associated genes including *MAGEA1*, *MAGEA12*, *IL8*, *FOXD1*, *IL1B*, *POSTN*, *PRAME*, *MMP9*, *SERPINE* and *CTGF*. Genes conserved between metastatic stages (n = 67) too holds several key regulators of melanoma like *VEGFA*, *GDF15*, *SPP1*, *UPP1*, *SPRY4*, *FGF2*, *CENPN*, *SERPINA3*, *BUB1*. The complete list of both gene sets along with its description is given in the S2 File. Despite heterogeneity in the conditions used to generate the datasets, the meta-analysis shows that these few biomarkers and known cancer linked genes show consistent upregulation, which not only statistically revalidates their significance and essential similarities in the dataset but also reveals novel melanoma associated genes.

To further validate the reliability of the study datasets and analysis results, we compared the fold change analysis of DE genes across different stages (Fig 2A). The average fold changes observed for CnM, CM and LN are ~0.4, ~0.5 and ~0.7, respectively. The total number of up-regulated genes in LN is also much higher than any of the other stages, as evident from the major drift in fold change volcano plot, whereas, CnM contains the least number of DE genes. Fold change analysis suggests a significant down-regulation of gene expression in cutaneous melanoma stages, especially during disease metastasis (S2 Fig). Closer investigation of the DE gene list for each stage revealed several melanoma biomarkers and associated genes. For example, the MAGE family genes (few of which are considered as melanoma biomarkers) and *IL8* (a well-known cancer related gene) are among the top-ranked genes enriched in our significantly up-regulated gene list [18–21]. Table 2 highlights the top 20 DE up-regulated genes in decreasing order of fold change in each stage. It is not a surprise that a known melanoma biomarker gene *PRAME* [22] is consistently found to be topmost up-regulated gene in all the melanoma stages. Among the MAGE family genes, *MAGEA12* is consistently highly up-regulated in all the melanoma stages, whereas *MAGEA1* is up-regulated only in the metastatic melanoma stages. CnM is also characterised by the expression of several keratin family genes involved in building structural framework of epithelial cells along with S100 family genes, involved in calcium binding and cellular immune responses. Apart from the genes mentioned above, several other genes that are known to be expressed in melanoma are also represented in the top-20 gene list, which includes *GDF-15*, *CDH2*, *CTGF*, *SERPINA3* and *POSTN*. Interestingly, *POSTN* (Periostin) is reported to play a role in accelerating melanoma metastasis via the integrin/mitogen-activated protein kinase (MAPK) signalling pathway [23].

**Table 2:** Differentially Expressed Genes (in the decreasing order of fold change).

CnM	CM	LN
*PRAME*	*PRAME*	*PRAME*
*S100A7*	*SPP1*	*SPP1*
*S100A8*	*IL8*	*IL8*
*S100A9*	*MAGEA12*	*MAGEA12*
*SPRR1B*	*FOXD1*	*POSTN*
*KRT14*	*POSTN*	*IGFBP3*
*KRT16*	*IGFBP3*	*TNC*
*KRT6B*	*MAGEA1*	*GJC1*
*POSTN*	*FAM198B*	*CTGF*
*FAM198B*	*SULF1*	*LILRB1*
*SERPINB3*	*IL13RA2*	*HLA-DRA*
*CXCL10*	*CTGF*	*IL13RA2*
*SERPINB4*	*GDF15*	*FAM198B*
*FOXD1*	*HAS2*	*SERPINA3*
*LPPR4*	*CDH2*	*EIF2S3*
*CXCL9*	*PLAT*	*ID3*
*CSTA*	*SNX10*	*CD74*
*IL8*	*CCL20*	*MAGEA1*
*KRT5*	*SDC3*	*SULF1*
*MAGEA12*	*IL1B*	*CCL5*

We also investigated the likely global changes due to DE genes, by performing gene ontology (GO) enrichment analysis (using a threshold p < 0.01). After the first transition, the most over-represented GO term corresponds to skin development and immune response (S3 Fig). However, as melanoma progresses, the representation of GO terms associated with blood vessel development and adhesion increases, whereas the representation of skin development related GO terms decreases. In LN, the most over-represented GO terms for up-regulated genes are immune response related processes and cell cycle.

In a nutshell, the DE analysis shows expected outcomes and from the large set of DE genes only a small number of genes are conserved, however the conserved gene set contains several known melanoma and cancer associated genes which validates the authenticity of our data processing procedure and quality of the processed data.

### Co-expression networks

For further analysis and prediction of novel dysregulated genes, four gene interaction maps were constructed using gene pair-wise Pearson Correlation Coefficient (PCC; see materials and methods). The reason for choosing PCC is that it is the most widely used method to identify the linear relationship between two random variables and captured significant relationships even when the genes have non-normal distribution [24]. We identified the correlation coefficient (***r***) distribution and also estimated the p-value and False Discovery Rate (FDR) associated with each correlation coefficient which is summarized in S4 Fig. Moreover, the analysis of free node count at different network thresholds helped us in deciding the reference network threshold, i.e. *r*> = 0.5 and FDR< = 0.05 (see S5 Fig).

Next, we evaluated the network qualities by comparing its topological properties with random networks of the same average degree and genes. Random networks were generated using Erdos-Renyi model [25], which creates every possible edge with equal probability '*p*', independent of other edges. The degree of a vertex in such a random graph follows a Poisson distribution with most nodes having approximately same number of links. Kolmogorov-Smirnov test [26], which evaluates the significance of deviation, suggest significant deviation of the inferred gene network from random networks (P<2.2e-16, Figure A in S6 Fig).

Generally in biological systems, a few genes have a significantly higher number of connections as compared to the other genes in the network i.e. they act as hub genes. Such scale free topology signifies the network robustness and its resistance to random gene knockout. Any scale free network is generally characterized by “heavy tailed” nature of node degree distribution (Figure B in S6 Fig) [27]. We observe a similar distribution of decreasing degree distribution with increasing links, marking the presence of genes with few connections to the 'hub' genes. We then performed a statistical measure to check scale free topology by testing power law distribution fit to each of the network model using bootstrapping hypothesis test [28]. We found that the Normal and CM networks do not significantly fit to a power law model (P<0.1), and hence the networks are not scale-free. However, “heavy tailed” degree distribution of the entire network and their deviation from the random networks provides the characteristic of a true complex biological network. Other fundamental network topological parameters are given in the Table 3. It is observed that data size has no significant impact on fundamental topological network properties with normal being the most connected network model and CnM, the least connected. Although a significant variability among the four network models exists, yet metastatic samples are closely related to each other owning to similar topological properties like edges, clustering coefficient and degree centralization. Overall, these topological properties suggest the differences in the expression or connectivity profiles of the same genes under variable conditions.

**Table 3:** Fundamental network properties.

	Normal	CnM	CM	LN
**Sample size**	97	183	246	116
**Edges**	4,269,800	368,136	1,605,642	1,835,739
**Unconnected nodes**	488	2406	2546	1228
**Clustering coefficient**	0.57	0.39	0.53	0.53
**Density**	0.04	0.004	0.15	0.02
**Centralization (degree)**	0.166	0.06	0.12	0.12

### Differential network analysis

**Fig 3:**
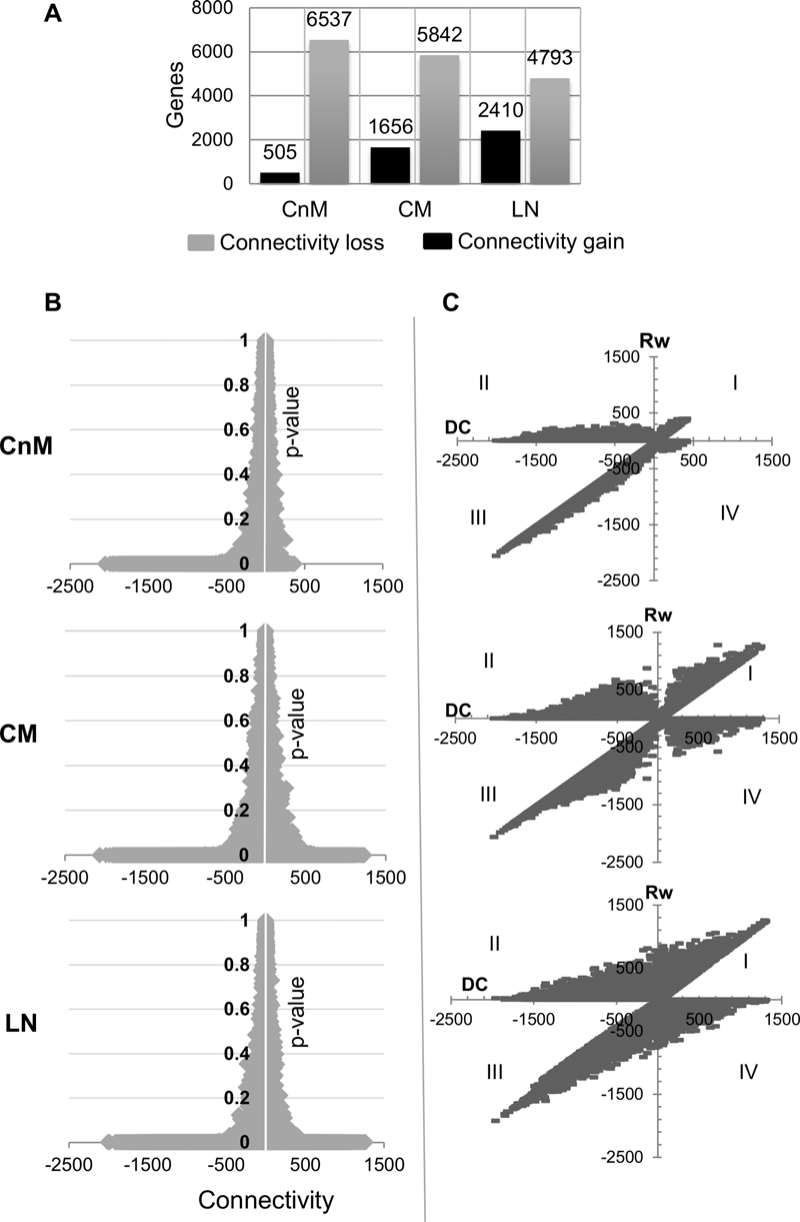
A. Number of genes significantly differentially connected (DC; either loss or gain) in each tumor stage with respect to the normal stage network. B. For each gene DC is plotted with its statistical significance (FDR adj.p-value) and with disease progression, number of genes with increased network connectivity increases under the significant p-value (p < 0.01) as revealed by horizontal line on the positive x-axis. C. For each gene, the calculated gained co-expression (Rw) is plotted with its overall DC score. The graph explains that with disease progression, genes tends to intensify the alterations in the connection types by gaining as well as loosing connections which may or may not alter its DC. Four quadrants suggest that genes may gain novel edges with increased DC (quadrant I) or gain novel connection despite losing overall DC (quadrant II) or lose edges with decreased DC (quadrant III) or show increased DC despite losing connections (quadrant IV). In the graph single gene was plotted two times to correlate Rw (+ve) versus DC and Rw (-ve) versus DC as both of the terms were found to be independent.

Most of the differential network analysis, not specific to melanoma, relies on the gene degree difference (Differential Connectivity; DC) across two or more networks to elucidate altered gene set. In order to study DC in melanoma genes, we measured each gene DC across different stages of melanoma with respect to the normal network and its statistical significance in terms of p-value and false discovery rate (adj.p or FDR adjusted p < 0.01). As expected, a significantly high degree loss is observed in different melanoma stages (Fig 3A). For each melanoma stage, more than a two-fold increase in number of genes with connectivity loss than gain exists. Analysing the differential connectivity as a function of p-value suggests that a majority of the genes with high differential connectivity have significantly low p-value (Fig 3B). The higher loss of connections with low p-value statistically confirms the edge loss from normal to cancer tissue. Also, the asymmetrical nature of the graphs suggests the presence of genes with a higher number of lost connections than gained. Both the metastatic stages are characterized by a nearly symmetrical graph, whereas the non-metastatic stage is asymmetric.

### Differential connectivity vs edges rewiring

We deciphered the altered connection types in each of the melanoma stages as compared to that in the normal, by overlapping the respective networks. In order to scrutinize the assumption whether edge rewiring produces different results than DC, we compared the results presented by the two approaches.

For each of the melanoma stages, we found a high number of genes with novel connections in-spite of the overall degree loss as compared to that in the control (Fig 3C; II quadrant). Each comparison is characterised by a diagonal relationship between connectivity loss and nodes lost (EL); connectivity gain and novel nodes gain (EG). The dense nature of the graph reflects high proportions of edge rewiring during metastasis, LN the most gene-rewired state. Thus, melanocyte to melanoma transformation begins with significant loss of network robustness, however, progression to metastatic transformation occurs only after multi-edge gains. We believe that the multi-edge gain, displayed by a gene set could be an important phenomenon for transformed biological activity and can help identification of genes in the most aberrantly rewired networks. Therefore, in the upcoming part of this manuscript, we will primarily focus on the shortlisted DC genes with significantly gained novel connections.

### Multi-edge rewiring in melanoma progression

**Fig 4:**
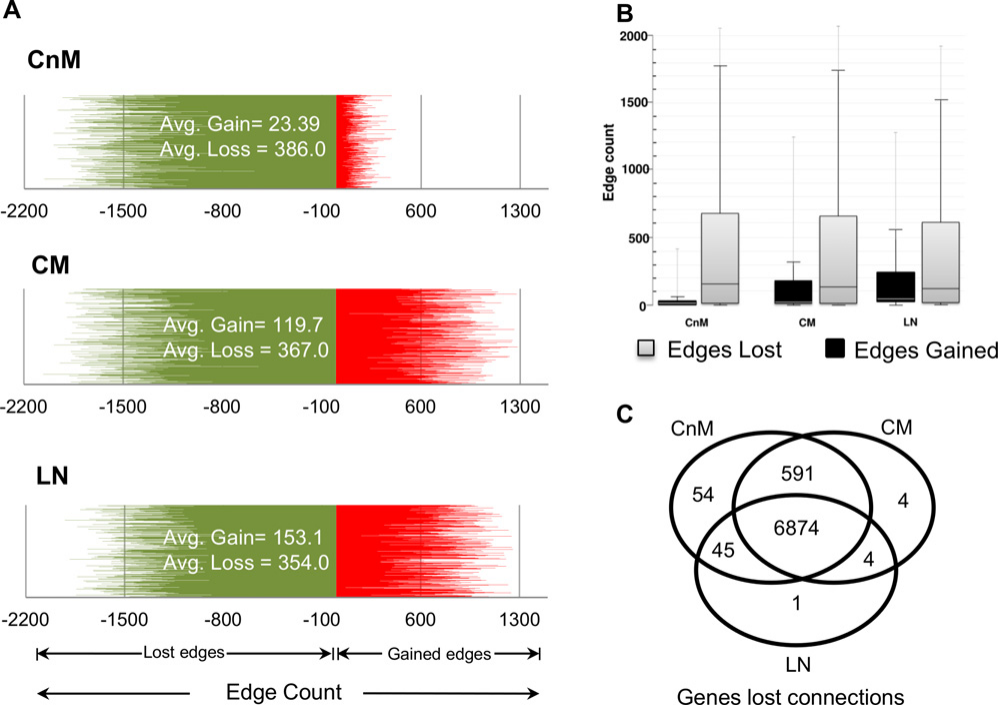
A. Line graph to observe the difference between edges lost (color: green) and gain (color: red) by a single gene in different melanoma stages. The observed trend suggests consistent and significant increase in novel edges gain, after the onset of metastasis. B. Boxplot showing the differences in the number of novel edges gained (color: red) and lost (color: green) by genes in each stage. C. Comparison for the genes that displayed connectivity loss (edge loss > 20) across different melanoma stages. Venn diagram suggests remarkable similarity of gene behavior across mutually exclusive datasets and loss of connections is a conserved phenomenon.

For elucidation of aberrantly networked genes, network connectivity rewiring analysis was performed for a stage-wise assessment of the edge rewiring. The analysis reveals that melanocyte to melanoma conversion initiates with a significant edges loss (highest EL = 2059) as compared to the number of edges gained (highest EG = 422). We observe a sudden increase in genes displaying EG, while maintaining its connectivity loss after the second transition (Fig 4A). Box-plot analysis clearly illustrates the effect of melanoma on gene connections, the number of edges lost is found to be as high as ~2000 in all the stages of melanoma (Fig 4B). In order to confirm if the genes with lost connections in CnM retain its connectivity profile in later stages, we compared the genes that lost more than 20 edges (arbitrarily selected value) across different stages of melanoma. More than 90% of the CnM genes that displayed EL continue to loose edges in CM and LN (Fig 4C). Thus, global analysis suggests that a large set of genes actively rewires its connection types, many of which gain novel connections and, if not significantly down-regulated, may actively be involved in aberrant networks in melanoma progression, especially in the cells programmed for metastasis.

A closer analysis of the results reveal that the gene that gained maximum edges during the first transition is *KIDINS220* (EG = 422, EL = 211) while the gene with the highest loss of connections is *C1orf216* (EG = 4, EL = 2059). During the non-metastatic to metastatic transition, the gene *LRP5* gained the highest number of connections (EG = 1283, EL = 113), while *C1orf216* continued to be the gene with the highest edge loss (EG = 0, EL = 2065). After the third transition, gene *ASNSD1* gained the highest number of edges (EG = 1254, EL = 32) whereas gene *ARMC9* (EG = 19, EL = 1924) lost maximum number of edges. However, the analysis of top five rewired genes do not list any known recognized melanoma marker, thus such genes may be explored as potential targets.

### Disease genes are significantly rewired

**Fig 5:**
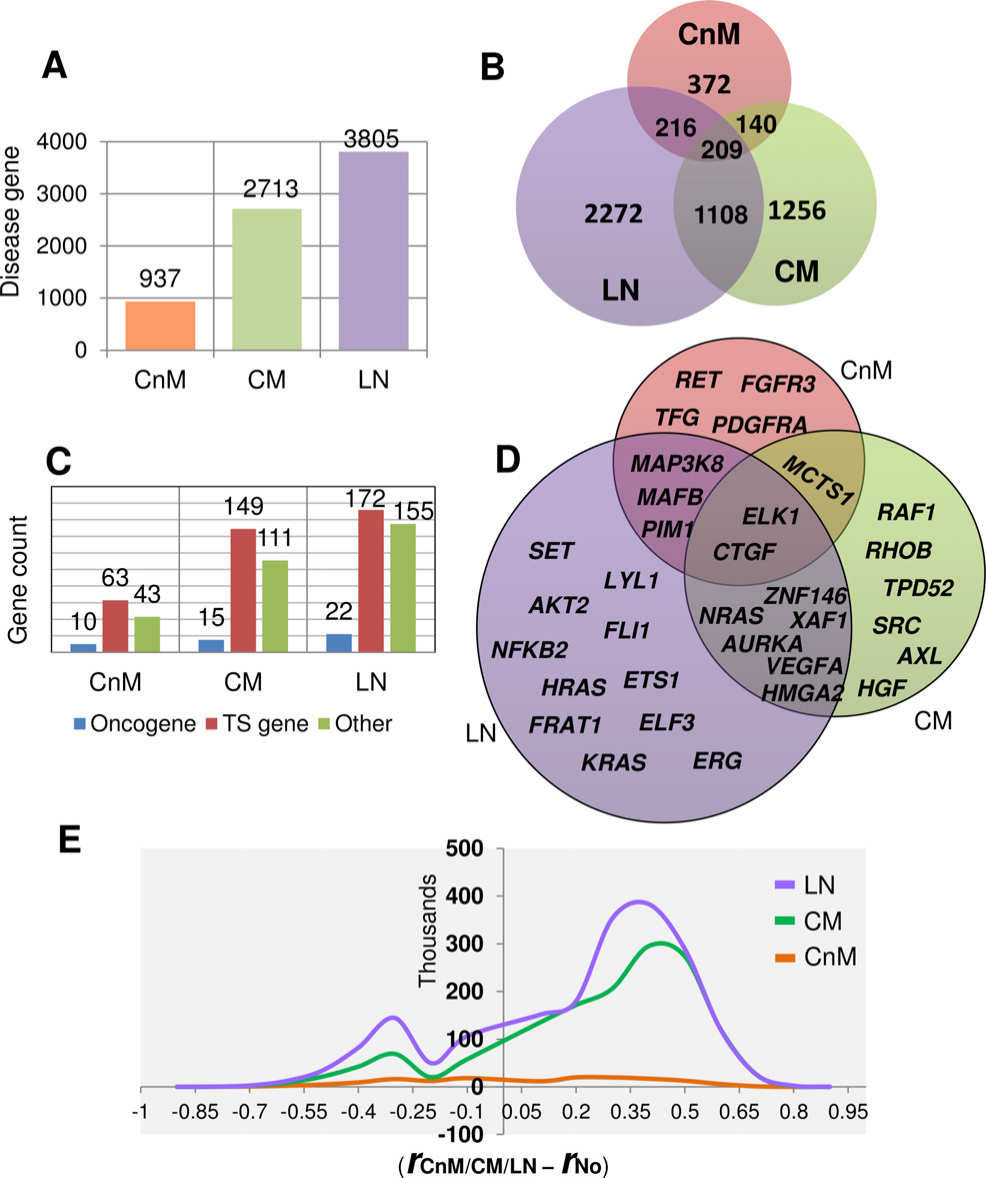
A. Total number of shortlisted putative disease genes with abnormal behavior (either up-regulated and/or network rewired) in each stage during melanoma progression. B. Number of shortlisted genes conserved across multiple stages which is comparatively many fold higher than DE genes alone. C. Number of different known tumor associated genes (tumor suppressor, oncogene and other tumor associated genes) identified among shortlisted disease genes for each stage. D. Oncogenes identified in each melanoma stage, the genes common in each stage are also displayed as intersections. E. Line graph showing the frequency of differential correlation coefficient observed for each edge between putative disease genes in each melanoma stage network versus control network. High value of differential correlation coefficient means high strength of association between disease genes in perturbed network as compared to normal.

By comparing the dataset of each stage with the normal dataset, we generated a gene list of disease genes that are either significantly upregulated or abnormally connected (see materials and methods). Our aim was to derive a novel gene set that could possibly account for stage-wise network aberrations during melanoma progression. Interestingly, the metastatic stages include a very high number of shortlisted genes (i.e. disease genes). The highest number of putative disease genes is found in LN as compared to that in other stages (Fig 5A–5E). It is evident that the high number of disease genes may help us to understand the metastatic behaviour of the genes in the dataset and identify novel associated biomarkers. It is found that 60% of the CnM genes retain its activity profile in one of the other two metastatic stages. We found that 59% of the uniquely stage specific disease genes in LN that maximally shares 1317 genes with CM. Interestingly, 209 disease genes are dysregulated across all the stages, including the genes which are not necessarily differentially expressed, presumably required for melanoma progression. To gain further insights, we investigated its GO terms and found that the over-represented processes are mainly related to immune response, cell activation and migration (data not shown). A closer investigation reveals that 91.15% (n = 762), 97.83% (n = 2520) and 89.56% (n = 2969) of the rewired genes in the disease gene list are not DE in CnM, CM and LN, respectively. Moreover, out of the 209 conserved genes, only 7 DE genes are rewired too. The seven genes are *IFI30*, *CXCL10*, *APBB1IP*, *POSTN*, *C1QA*, *MMP9* and *GBP1*. The results suggest that cancer genes in melanoma mainly modulate its activity either by changing its expression or the expression of other genes. We also found that few well-known melanoma biomarkers like *NRAS*, *CDK4* and *CCND1* are significantly rewired in different metastatic melanoma stages, even if not differentially expressed (in the particular stage). For example *NRAS* is not differentially expressed in CM (logFC = 0.8), yet it is rewired with an edge gain of 234.

Although, different oncogenes and tumour suppressor genes may exert opposing roles in different forms of cancer, yet in order to elucidate novel cancer associated genes, we searched disease gene list for known human cancer related genes. Not surprisingly, cancer related genes are found to show stage-wise amplified aberration in CnM (136 genes), CM (275 genes) and LN (349 genes). In all the stages, alteration of tumour-associated genes seems to be a necessary phenomenon. We collected annotated cancer associated genes from several databases [29–31] and classified our disease gene lists into three categories: oncogenes, tumour suppressors and other tumour associated genes. We found a high number of tumour suppressor and other tumour associated genes than oncogenes, showing aberrant behaviour in each of the melanoma stages (Fig 5C). The Fig 5D shows different oncogenes predicted to gain alteration with the stage wise progression of melanoma. The list comprises of several well-known melanoma-associated-oncogenes including *RAF1*, *AURKA* and *SRC* along with putative novel oncogenes. Interestingly, *CTGF* gene conserved in all the three stages, plays an important role in cell adhesion, migration, proliferation, angiogenesis and recently reported to be a therapeutic target against malignant melanoma [32]. Another oncogene *MCTS1* is preserved in both the stages of cutaneous melanoma and surprisingly the gene product is under clinical trials as a drug target for small cell lung cancer [33]. The oncogenic potential of most of these genes is already well recognized (e.g. *SRC*, *NRAS*, *VEGFA*, *NFKB2* etc.), this validates our findings and the dataset as well as suggests that the other genes in the list for example *MCTS1*, *ELK1* and *ZNF146* may be further investigated for its potential role in melanoma metastasis.

To further elucidate the disease linked properties of the disease gene list; we compared the differential correlation strength (*r*_*d*_ − *r*_*c*_) of disease gene edges in the disease and normal stages (Fig 5E). The differential analysis suggests that melanoma linked genes strongly communicate, especially in metastasis, with an edge strength difference as high as ≥0.9. We observe that the maximum number of genes generated differences in edge strengths ≥0.4, which is statistically significant for a large dataset.

### Identification of integrated pathway clusters

**Fig 6:**
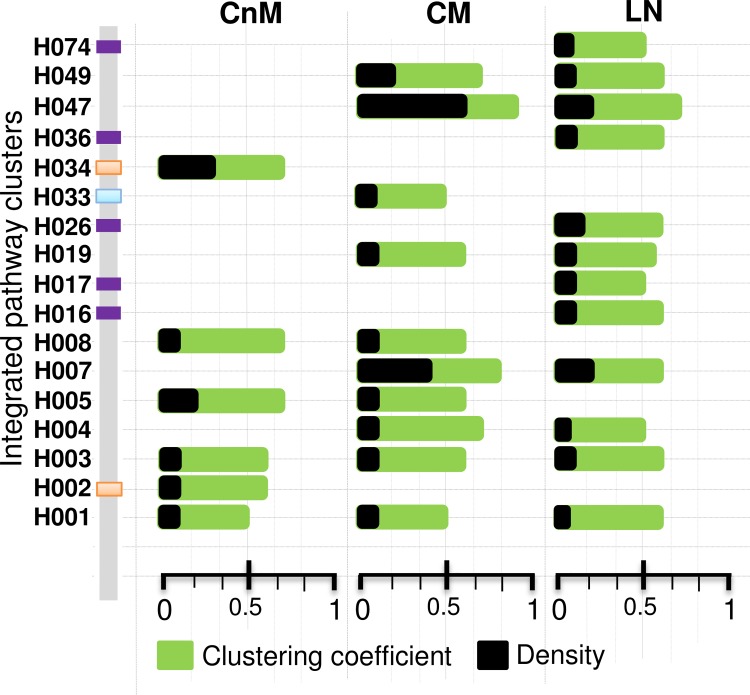
Predicted IPCs identified in each melanoma stage along with the two fundamental network properties (clustering coefficient and density) of its enriched genes. The colored strip represents stage specific IPCs; orange is CnM specific, grenn is CM specific and voilet is LN specific. More information about the IPCs is available in S3 File.

To gain insight into the functional themes and its qualitative drift during each transition of melanoma progression, we scanned the shortlisted disease gene list for different “integrated pathway clusters (IPCs)” (adj.p < 0.05) for each stage. IPC is a large gene set build upon a particular biological theme and comprised of several sub-clusters, each representing a particular biological pathway which may or may not be overlapping [34]. For each of the disease stage we predicted several IPCs composed of disease genes and investigated its respective fundamental network properties (Fig 6). Interestingly, we observe several stage-specific IPCs along with IPCs preserved across two consecutive conditions, whereas only two IPCs are common across all the three stages of melanoma. The stage-wise information about different IPC IDs, associated genes and adjusted p-value is given in the S3 File.

For cutaneous melanoma IPCs “H003”, “H005” and “H008”, which corresponds to “Cell adhesion molecules”, “Extracellular matrix (ECM) organization” and “Cellular responses to stress”, respectively, are aberrant in both the stages of cutaneous melanoma but absent in LN. In CnM, the cluster “H003” (n = 98, p = 7.70E-009) contains genes that allow cells to interact its surrounding environment like *CDH1*, *CNTN1*, *CD2* and *CLDN7*, which increases by two-fold in number after metastatic transition (n = 193; p = 4.3E-004). Cluster “H005” contains 63 genes (p = 2.5E-006) in CnM and 142 genes in CM (p = 3.8E-008) including multiple collagen, thrombospondin 1 (*THBS1*), MMP family genes along with few cell signalling receptor genes like *TGFBI*. We found two CnM specific clusters, i.e. “H002” and “H034” that corresponds to “hemostasis” (n = 185; p = 9.2E-003) and “Amoebiasis” (n = 24, p = 9.9E-004) respectively.

After metastasis, the cluster related to cell cycle (H007; n = 202, p = 4.12E-014) displays its appearance, marking its importance for metastatic growth, and continues to thrive in LN (n = 254, p = 2.0E-013). Several new IPCs emerge in CM that is retained in LN too (70% of CM), indicating its importance in promoting metastasis. Apart from the cell cycle, the cluster corresponding to PLK1 signalling (H047; n = 30, p = 3.4E-004) (an important signalling pathway mediated by “polo like kinase” in a wide range of cancers [35]) displays exceptionally high networked properties unique to CM. This cluster contains *AURKA*, *BUB1*, *CDK1*, *CDCA8* and other genes known to be associated with cell cycle related processes. Interestingly, the cluster “H033” (n = 29, p = 0.04) is uniquely expressed during CM and represents “HIF-1-alpha transcription factor network”, a pathway expressed in cells thriving under high metabolic conditions or low oxygen concentration (i.e. hypoxia) and thus potentially corresponds to the angiogenesis related events in cutaneous metastasis.

After the final transition from CM to LN, 70% of the CM IPCs are retained; however, four new IPCs corresponding to various cellular events emerge in LN. The new IPCs include “H016” (n = 186, p = 0.0015), “H017” (n = 79, p = 0.02), “H026” (n = 118, p = 0.003) and “H036” (n = 121, p = 0.001) which corresponds to “chromatin modifying enzymes”, “Signalling by NOTCH”, “Organelle biogenesis and maintenance” and “Endocytosis” respectively. Interestingly, “H019” a cluster that corresponds to immune related processes shows exceptionally higher statistical significance in LN (n = 244, p = 2.6E-011) as compared to that in CM (n = 141, p = 0.02), despite a high increment in corresponding gene counts. Another immune related or cancer related cluster identified by “H001” is conserved across all the three stages of melanoma, with conserved network properties and gene count.

Summarily, the IPC themes covered a wide range of processes essential for cancer progression, including IPCs related to immune system, signalling events, cell cycle related process, cell adhesion and ECM organization. We found several unique stages specific as well as conserved IPCs composed of DE as well as rewired genes. We found several cancer related IPCs and also investigated the connectivity profile of clusters in respective networks. The enriched list of cancer related processes and networked genes potentially contain novel markers or biological associations.

### IPC pathways distribution analyses reveal dysregulated metastatic melanoma pathways

**Fig 7:**
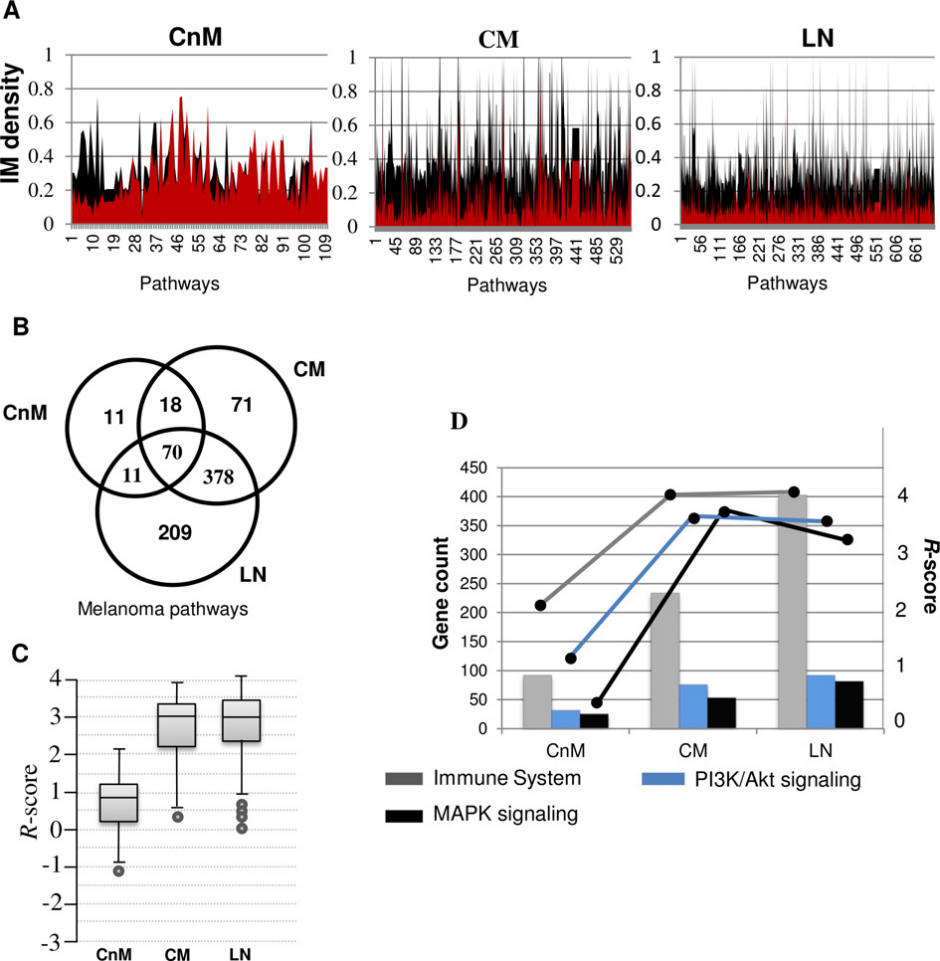
A. Comparison of Intra-Modular (IM) sub-network density for every over-represented pathway in each melanoma stage (color: black) vs normal stage (color: red). It may be observed that the density of connections among disease genes in enriched sub-networks, generated from normal stage, decreases with disease progression. B. Conservation of enriched pathways from disease genes across different melanoma stages. C. Box-plot representing range of calculated *R*-scores for every pathway in each stage. High *R*-score implies higher dysregulation in the connectivity pattern of pathway disease genes in perturbed network as compared to the normal network. D. A combined bar and line graph representing gene count and calculated *R*-score of the three most common melanoma associated pathways. MAPK pathway decreased *R*-score is lowered after CM to LN transition despite increase in enriched gene count, whereas, other two pathways show consistent *R*-score irrespective of gene count.

We also analysed the distribution of pathway components within each theme. We measured the intra-disease gene connectivity profile of each pathway members (n > 5, density > 0, clustering coefficient > 0) and analysed its differential network properties with normal (control). Our primary aim in this analysis was to elucidate the pathways that can act as a better representative of a particular metastatic stage by prioritizing each pathway term using significantly deviating network properties with respect to that in the normal. There are 110, 537 and 668 pathway clusters predicted in the three stages in the order of melanoma stage progression. There are 70 different pathways that are perturbed across all the stages of melanoma, corresponding to diverse cellular events, including pathways like MAPK and PI3K/Akt signalling. However, the pathway gene set generated from our “unbiased” approach represents a crude picture of gene expression which must be processed for more meaningful global phenotypic inference, as suggested by Markowetz et al. [36]. Therefore, for each of the pathway, we measured the gene-gene association by calculating its intra-modular (IM) density (Fig 7A). Comparative analysis of IM density reveals strong differences in gene associations for a given pathway in each perturbed network as compared to the normal stage network. Once melanoma metastasizes, we observe a sudden increase in the number of predicted pathways, along with its gene count and IM density. As expected, the connectivity profile of disease gene set in each pathway tends to decrease with melanoma progression as revealed by IM density comparison. We also observed that 90% of CnM pathways are also found in one of the other metastatic stages of melanoma, whereas 83.4% of CM pathways are retained after metastasizing to LN (Fig 7B).

Although IM density helps in discovery of the most densely connected pathways, however we found that only IM density was not sufficient to fetch the most significantly rewired pathways. Hence we calculated pathway connectivity (PC and pathways sharing members; discussed later), total connectivity and module density for each pathway. For each sub-cluster, these network properties were compared with that of the previous stage (control) by mapping the same gene set onto respective weighted co-expression networks to generate a pathway specific relevance score (*R*-score; see materials and methods). The calculated *R-*score for each pathway helped in prioritizing the most active pathways that are significantly altered or rewired independent of gene counts, as compared to that in the normal stage. The comparison of *R-*scores clearly reveals the distinct nature of dysregulated pathways in melanoma metastasis as compared to that in the non-metastatic stage. In order to gain better understanding, we compared *R-*score and gene count fluctuations of three well-established melanoma associated pathways conserved across the three stages of melanoma (Fig 7D). There is a sudden increase in cellular response during non-metastatic to metastatic transition for all the three pathways. *R-*score analysis represented in the Fig 7D suggests that there is an insignificant alteration of “MAPK signalling pathway” during nodal metastasis as compared to the other two pathways, despite consistent increase in its gene count. For “immune response” and “PI3K/Akt signalling”, comparable increase in activity is observed after each transition.

### Metastatic pathways prioritization using network properties

**Fig 8:**
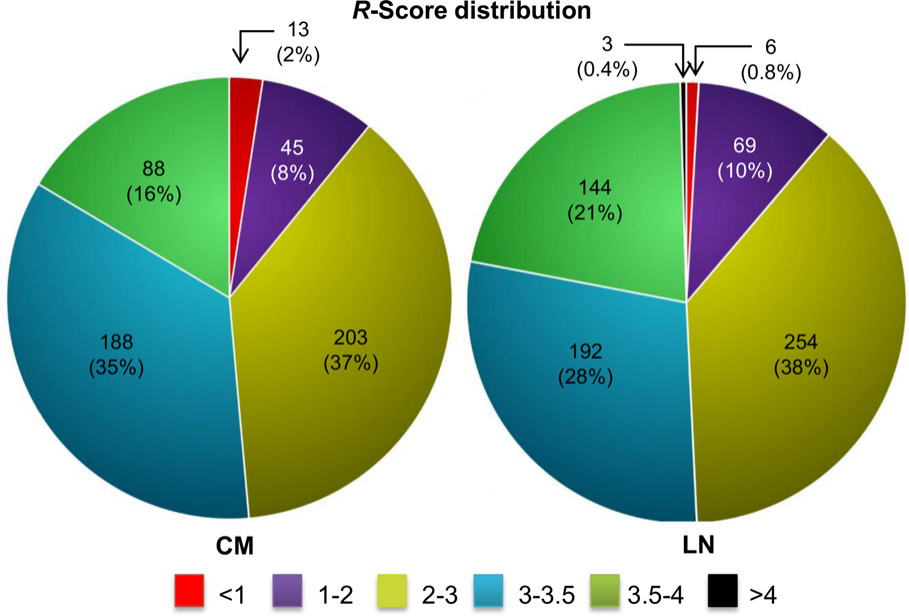
Pie chart representing *R*-score distribution of all the enriched pathways in each metastatic melanoma stage.

Our primary interest was to elucidate the most aberrant events from the perturbed networks of melanoma metastasis. *R-*score distribution analysis helps in classifying the pathways list in accordance to closeness with each other and deviation from normal set (Fig 8). We observe ~50% of the pathways have *R* > 3.0 in both the metastatic networks, which includes known melanoma-associated pathways. For instance, pathways like “cell cycle”, “signalling by WNT in cancer”, “PI3K/Akt signalling”, “EGFR signalling in cancer” and “MAPK signalling” have *R* > 3.50 in both the metastatic networks. The small enrichment of pathways with high *R-*score (>3.50) and its close association with the disease further highlights the relevance of *R-*score.

**Fig 9:**
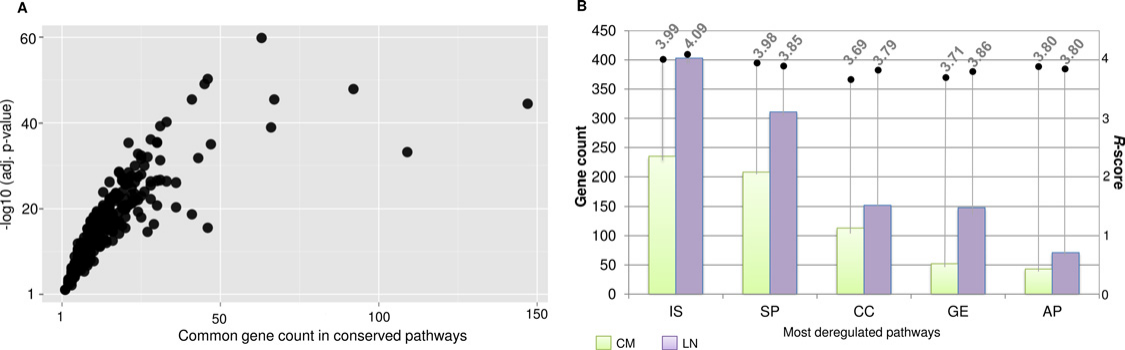
A. Scatter plot representing the statistical significance of gene composition similarity in pathways conserved in both melanoma stage. Log transformed fishers exact p-value is plotted against the common number of genes of a pathway commonly enriched within two different metastatic melanoma stages. B. Bar graph representing the gene count enriched for top 5 (on the basis of *R*-score represented on the right axis) conserved within metastatic stages. Here, IS is “Immune System”; SP is “Signalling Pathway”; CC is “Cell Cycle”; GE is “Gene Expression”; AP is “Apoptosis”.

A comparison of the CM and LN pathway lists reveals that 448 pathways are altered in both the perturbed networks. We measured the statistical significance of the reproducible pathways by calculating gene set similarity for each pathway in both the stages. We observed a high statistical similarity (Fischer’s exact test FDR adj.p < 0.05) amongst all the gene sets of conserved pathways across both the metastatic conditions (Fig 9A). These inferences of high pathway similarity, thus creates an opportunity for deducing pathway dependencies in metastatic melanoma. *R-*score analyses reveals the most altered set of events that broadly include pathways related to “immune system”, “gene expression”, “cell signalling”, “apoptosis” and “cell cycle” (Fig 9B). Quite expectedly, the immune system related processes quantitatively exceeds in all the aspects of the defined properties. Strikingly, we also observed the enrichment of limited numbers of other disease related pathways, for instance HIV infection or prostate cancer. Such significantly high *R*-scores indicate quantifiable characteristics of genes like having multi-disease association and most importantly help understand disease mechanisms. The complete list of pathways comprises of several different cellular events involved in melanoma metastasis, the aberration of which increases with metastatic progression.

### Signalling pathways in metastatic melanoma

Emerging knowledge about several melanoma variations (e.g. oncogenic mutations) advanced our understanding about the signalling events involved in melanoma. However, complex interplay between these events is still a least studied phenomenon. Therefore, in order to assess the complex relationship between various signalling events and elucidate the most frequent cross-talk event, we scanned the enriched pathways lists for various signalling events.

**Fig 10:**
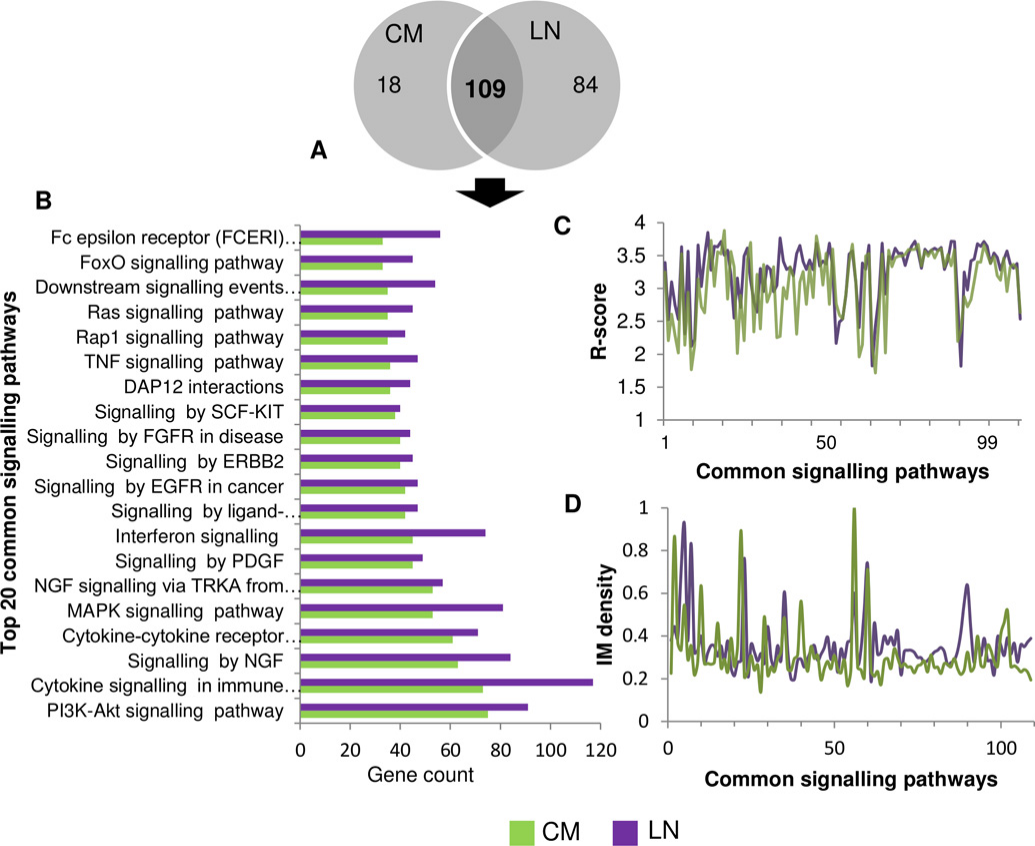
A. Total number of conserved and predicted signalling pathways in each metastatic melanoma stage. B. Top 20 conserved signalling pathways of CM with their gene count in both the metastatic stages. C. Comparison of *R*-score trend observed for the conserved signalling pathways. It can be observed that conserved signalling pathways possess similar *R*-score across mutually exclusive datasets. D. Peaks representing IM density of conserved signalling pathways, follows similar pattern in both the metastatic melanoma stages.

The numbers of signalling pathways predicted in CM and LN are 127 and 193, respectively. Out of these, 85.8% CM signalling pathways are retained in LN (Fig 10A and 10B). After metastasis, the signalling pathway that displays the most aberrant behaviour are related to “B-cell signalling” (n = 36, *R* = 3.88), whereas, in LN “Cytokine Signalling in Immune system” (n = 118, *R* = 3.84) generate the highest *R* score. In terms of gene count, one of the largest observed clusters is associated with the “PI3K-Akt signalling pathway” (n^CM^ = 76, n^LN^ = 92) in both the metastatic melanoma stages, which plays an essential role in melanoma and progression in other cancers too. We also observed several other recognized oncogenic signalling pathways commonly found in both the stages of metastatic melanoma. Among these pathways, “MAPK signalling”, “signalling by EGFR in cancer”, “PI3K/Akt signalling”, “signalling by SCF-KIT”, “signalling by hedgehog” are the top ranked (amongst top 20; R > 3.5) in both the metastatic melanoma stages. The closeness of *R-*score and progressively increasing gene count among these top-ranked melanoma associated pathways, indicates its cooperative efforts in maintaining the complexity of disease during melanoma progression. Moreover, the network density and *R*-score of the common pathway terms follow a similar trend in both the mutually exclusive datasets (Fig 10C and 10D), which also reveals the conserved behaviour not deduced by any traditional expression based analysis.

### Cross-talks of signalling pathways

For efficient drug development for a desired therapeutic action, an understanding of the coordinated action of different signalling pathways is essential. Further investigation was carried out by elucidating the statistically significant co-members between two different pathway gene sets (PC > 1, n > 5, Fisher’s exact test adj.p < 0.05), which directly leads to an indication of pathway cross-talk. This pathway linkage network revealed several important highly connected signalling pathways in each metastatic stage.

We observe a higher number of probable signalling cross-talk events (sigPC) for selected pathways in LN as compared to that in CM, presumably positively correlated to the disease complexity (see S4 File). Surprisingly, “signalling by NGF”, for cell invasion and migration during metastasis, putatively cross-talks with the maximum number of signalling pathways (sigPC^CM^ = 73, sigPC^LN^ = 80), followed by “cytokine signalling in immune system” (sigPC^CM^ = 71, sigPC^LN^ = 79). Among the other events, angiogenic pathways like “Signaling by FGFR in disease” (sigPC^CM^ = 63, sigPC^LN^ = 67) and “signalling by PDGF” (sigPC^CM^ = 59, sigPC^LN^ = 67) are also listed among the highly connected pathways in metastasis, possibly fulfilling the vascular requirements of metastasizing melanoma. Among the well-documented melanoma associated pathways, PI3K/Akt signalling (sigPC^CM^ = 58, sigPC^LN^ = 76) along with MAPK signalling (sigPC^CM^ = 44, sigPC^LN^ = 58) and EGFR signalling (sigPC^CM^ = 49, sigPC^LN^ = 59) too shares genes with a significant number of pathways. Apart from the aforementioned cellular events, a common feature in both metastatic stages is the presence of highly connected immune response, especially signalling by interleukins (sigPC^CM^ = 50, sigPC^LN^ = 69).

Next, we set out to find how the cell signalling pathways are inter-connected at gene level. In CM, NGF signalling genes display a dense nature of co-membership with other signalling events and maximally co-membered (n = 37) with “signalling by FGFR in disease”, “signalling by EGFR in cancer” and “signalling by PDGF”. Intriguingly, investigation of the cross-talk genes revealed several known melanoma associated biomarkers, including *NRAS*, *PTEN*, *SRC* and *RAF1*. The occurrences of the biomarkers together in multiple cross-talk events provide the necessary explanation of its potential anti-melanoma drug target activity. Several other genes may also be involved as mediator of signal from one pathway to another. Therefore we also investigated the cross-talk potential of each gene by calculating its occurrence in different signalling pathways (i.e. cross-talk frequency (*cf)*, see S4 File). *PIK3CB* and *PIK3R2*, PI3K signalling mediators, are genes involved in the highest number of signalling cross-talks (*cf* = 46). The relatively high cross-talk frequency of these genes highlights the exceptional role of PI3K signalling in metastatic melanoma and suggests a reason of targeting it for anti-melanoma activity. Next three genes in the order of decreasing cross-talk frequency are *NRAS* (*cf* = 41), *RAF1* (*cf* = 38) and *PTPN11* (*cf* = 38)*-* the genes are known for its aberrant behaviour in melanoma. We find 39 genes with *cf*> 10, which can be explored further, in isolation or in combination, for viability as drug targets.

Similarly, in LN we observe the strongest co-membership between “Cytokine Signalling in Immune system” and “Signalling by Interleukins” as well as “signalling by interferon”. However, “cytokine signalling in immune system” represents a general term for interferons and interleukins and hence the obvious co-membership. After ignoring this cross-talk, the next best cross-talk in terms of gene count is between “signalling by NGF” with the same set of pathways as in CM i.e. “signalling by FGFR in disease”, “signalling by EGFR in cancer” and “signalling by PDGF”. However, the intensity in terms of gene counts increases in LN than CM. In LN, 44 different genes are observed, out of which 17 cross-talk genes are common in CM too, which includes important genes like *NRAS*, *PTEN* and *CDK1*. The observed 27 new cross-talk genes in this multi-pathway cross-talk event in LN include cancer related genes like *AKT1*, *AKT2*, *MAPK3*, *KRAS*, *JAK1* and *JAK2*. Investigation of cross-talk potential elucidates 53 different genes with *cf* > 10, out of which only 19 genes are common with CM. In LN, *MAPK3* (*cf* = 53), *PIK3R1* (*cf* = 51), *HRAS* (*cf* = 46), *PIK3R2* (*cf* = 45) *and KRAS* (*cf* = 44) are the top 5 cross-talk genes, and as expected most of these are already recognized melanoma markers.

### Other pathways in metastatic melanoma

Apart from signalling pathways several other events can control cell cycle machinery, especially the ones related to cell cycle, activated in response to genomic instability or DNA damage [37]. 218 and 217 different pathway terms are co-membered with cell cycle pathway with significant number of common genes (fishers exact test p < 0.05) in CM (cell cycle *R* = 3.69) and LN (cell cycle *R* = 3.79), respectively. The connected pathway list includes diverse set of events including processes related to signalling pathways, tumour suppressors and cell cycle related events. The *R-*score analysis (*R* > 3.5) gives a clue about the highly perturbed cell cycle related gene sets intrinsic to the tumour cells. Among these, we observe several pathways that allow cells to skip its checkpoints and mediate its unimpeded progression especially via G1 checkpoint to S-phase. Interestingly, anaphase promoting complex (APC) mediated degradation events display exceptionally high connectivity by small gene count (n^CM^ = 13, *R* ^CM^ = 3.70, n^LN^ = 32, *R* ^LN^ = 3.63). The pathway is composed of several proteasomes and mediates the programmed degradation of tumour suppressor and cell cycle regulator genes. Majority of the shortlisted pathways, for instance- p53 mediated pathways, G1/S transition, G1/S checkpoint are already well documented for aiding uncontrolled proliferation in different cancer types.

Apart from the aforementioned events, our pathway list comprises a variety of cancer related events. For instance, “Apoptosis” (n^CM^ = 43, *R* ^CM^ = 3.80, n^LN^ = 71, *R* ^LN^ = 3.80), “Proteoglycans in cancer” (n^CM^ = 51, *R* ^CM^ = 3.56, n^LN^ = 47, *R* ^LN^ = 3.36), “Disease” (n^CM^ = 79, *R* ^CM^ = 3.66, n^LN^ = 288, *R* ^LN^ = 4.02), “Pathways in cancer” (n^CM^ = 86, *R* ^CM^ = 3.66, n^LN^ = 111, *R* ^LN^ = 3.68), “Hemostasis” (n^CM^ = 83, *R* ^CM^ = 3.39, n^LN^ = 116, *R* ^LN^ = 3.74), “cellular senescence” (n^CM^ = 40, *R* ^CM^ = 3.41, n^LN^ = 27, *R* ^LN^ = 3.11), “melanoma” (n^CM^ = 17, *R* ^CM^ = 3.41) are all enriched with a different set of genes and significant *R-*score. Intriguingly, we observe an exceptionally high surge in gene count and *R-*score for the pathway term “Disease”. Moreover, the IM density of the 17 genes in melanoma pathway in normal network is 0, whereas in CM it is 0.28. Such findings indicate the essentiality of synergistic action of diseases related gene circuit rewiring with gene upregulation, in driving the disease progression.

### Intra-modular hub gene druggability analysis

Next, we investigated the gene level network properties by identification of representative genes in each pathway. A representative gene (or hub gene) is centrally located and displays highest connectivity among the disease genes, therefore, potentially exploitable as a biomarker or therapeutic drug target. 99 hub genes in CM and 142 in LN are found in one or more pathways, and show remarkable aberrations in connectivity profile across both the metastatic stages. We also observed that only 4 hub genes (*MAPK9*, *RB1*, *CHUK* and *HDAC2*) are conserved across both the stages representing underlying heterogeneity among different stages. The associated rewiring dissimilarities amongst the regulatory genes of conserved pathways suggest stage specific regulation. To identify potential drug targets amongst the rewired genes, we performed druggable genome analysis of the hub genes using DGidb webserver, which searches existing collections of known or potential drug-gene interactions. (It includes over 40,000 genes and 10,000 drugs involved in over 15,000 drug-gene interactions) [38]. We found 22 hub genes in CM and 19 genes in LN that are (or its gene product) already known to have anti-cancer drug interactions. Further, we attempted to reproduce the results by submitting the hub gene list to CancerResource [39], a comprehensive knowledgebase of validated or curated gene-drug interactions especially for cancer dataset. Interestingly, 68 CM and 65 LN genes code for the products that specifically interact with at least one listed drug. Summarily, we are able to obtain 371 cancer relevant drugs targeting 129 different metastatic hub genes. Thus, 54.43% of the hub genes or its products are interacting with at least one cancer-related drug, which not only reaffirms the robustness of our analysis results but also confirms the presence of common features of cancer relevant genes across multiple cancer types. The analysis helped shortlisting cancer relevant drugs, which includes several compounds with known anti-melanoma activity while for others, the potency or efficacy still remains to be experimentally explored.

## Discussion

The study utilizes melanoma differential expression and network-based analysis to show that the traditional differential connectivity based analysis may miss several potential biomarkers, which can only be elucidated via alternate methods like network rewiring analysis. Moreover, the re-wiring analysis also overcomes the drawbacks associated with DE based analysis and the short-listed genes are several times conserved, even in the heterogeneous datasets.

We started our analysis by determining DE genes, which is in agreement with the previous findings, and we observed only a limited number of DE genes in heterogeneous datasets despite the low fold change threshold (logFC > 1). However it is observed large number of genes demonstrated similar network connectivity behaviour in perturbed networks without any changes in the expression profile as compared to that in the control dataset. As expected, the control network loses its robustness resulting in connectivity loss of a large proportion of genes. However, we observed that genes could gain a significant number of novel edges to get rewired, despite showing overall connectivity loss in the perturbed networks as compared to that in control, which may reflect its continued but disrupted effectiveness. These rewired genes along with the up regulated genes are much more strongly or uniquely connected to each other in the perturbed networks as compared to that in the control network, which resembles the properties of disease genes [40, 41]. Moreover, the correlation strength of the conserved edges amplifies with disease progression.

To uncover the oncogenic potential of shortlisted disease genes, we scanned the genes for the presence of known oncogenes. We found very limited number of oncogenes (~10–20) in each melanoma stage as compared to the number of other cancer related genes. This observation suggests that only a few oncogenes consistently alter their activity to drive malignancy. Moreover, the other possible explanation of this heterogeneity lies within the variety of genetic or epigenetic alterations that activates specific lineage of oncogenes that drive malignancy. Apart from this, we also observed significant disruption in the connectivity profile of tumor suppressors and other cancer related genes.

It is known that the gene set or pathway based analysis is better approach for determination of novel therapeutic targets, as compared to gene based analysis [15]. Therefore, the above findings further motivated us to functionally cluster the disease genes in each stage, which surprisingly revealed several cancer and related pathway IPCs. We observed the emergence of new clusters with metastatic progression, whereby CM acting as a connecting link between CnM and LN. Melanoma begins with appearance of biological processes like haemostasis, coagulation cascade and other cancer related biological processes. The comparison revealed aberration in pathways related to cell adhesion molecule and ECM organisation preserved in both cutaneous stage but absent in LN. This suggests that the skin melanoma cells require organization of structural scaffold for cell migration, possibly achieved by regulating epithelial topology. Hence therapeutic targeting of these pathways as early as possible in disease progression is potentially a more effective strategy, as the results of therapies are very poor in the late stages. Metastatic programing leads to the activation of pathways that corresponds to cell cycle progression aided by signalling events like PLK1 signalling that can mediate neoplastic transformation via initiation of early trigger for G2/M transition. Genes related to immune response pathways are consistent in all the stages, however in LN immune response related processes demonstrate sudden enhancement of alteration along with other important processes like cell cycle, RNA processing. Overall, the IPC analysis summarizes the first line of evidence that melanoma progresses with the increase in functional complexity and connectivity strength among a subset of genes, making it difficult for therapeutic targeting.

The information provided by IPCs analysis, however, did not specify the underneath biological processes that could be of any therapeutic relevance. Therefore, we split each IPC into its corresponding pathway components. Next we exploited the differential network properties of these clusters for stage specific pathway prioritization. Comparative analysis revealed that different melanoma stages possess several common pathways. Intriguingly, 83.4% of the CM pathways are statistically conserved in both metastatic melanoma stages. Expectedly most of the pathways that occur in each stage were melanoma or cancer causing and covers wide range of oncogenic events, e.g. immune response, cell cycle, apoptosis, hypoxia related events, signalling pathways in cancer including EGFR signalling, PI3K/Akt signalling, MAPK signalling, WNT signalling and other related pathways. The notable feature of the outcome was the sharp increase in pathway count and corresponding sub-network properties after non-metastatic to metastatic transition, which we believe is the regulated necessity of the cells programmed for metastasis.

In addition, for each of the pathway, the comparison of network property deviations in disease network as compared to that in the control highlights the functional significance of each pathway reflected by normalized *R-*scores. The relevance of a gene set was interpreted in context to the disease phenotype by the number of roles a gene set can play in different events. Not surprisingly, most of the well-known melanoma associated pathways have exceptionally high *R*-scores (*R* > 3.0). The comparative analysis of the results from different stages revealed consistent increase in defined properties and functional robustness of the predicted gene sets with melanoma progression. This novel network based prioritization of the shortlisted pathways can be exploited for stage specific therapeutic targeting of melanoma.

We know that the pathways are not static or isolated entity rather its dynamic properties allow a gene set to play a significant role in multiple and diverse events. Hence, we exploited this property for refinement of the pathway list by calculating statistically significant inter-pathway co-membership and gene-gene connectivity along with intra-pathway connectivity. Our analysis took the results a step further by investigating the co-membership attribute for prediction of the important signalling cross-talk and associated gene sets. We observed that p75 and trk family receptor mediated events, i.e. signalling by NGF, have genes with very high potential to cross-talk with other cancer related events. The pathway is very well documented to play functionally vital role in cell migration and invasion during melanoma tumorigenesis and therefore possesses high R-score in our analysis (*R* ^CM^ = 3.58; *R* ^LN^ = 3.58) [42]. Apart from this, several other melanoma-linked pathways like PI3K/Akt, MAPK signalling were also found to cross-talk variety of pathways. Interestingly the number of cross-talks and the gene strength amplifies with metastatic progression. This kind of information cannot be easily obtained from traditional network module based analysis, in which, connected gene set is treated as an isolated entity.

In the last phase of our analysis, we identified the highly connected hub genes that uncovered the variable co-expression relationships within same pathway across different stages of metastasizing melanoma. The little intersection of hub genes suggests (n = 4) its underlying heterogeneity and emergence of novel role as stage specific regulators, which leads to differential regulation of pathways in multiple cancer stages. To gain deeper insight we performed druggable genome analysis of the highest connected genes and predicted ~58% of hub genes known to play cancer related activity, thus rest of the genes may be potentially important for cancer related activity but requires further investigations. Hence it is important to perform pathway centric investigation for therapeutic targeting as suggested by Kreeger *et al*. [15]. Although the hub gene analysis did not play any role in proposing the underlying similarity amongst melanoma datasets, yet it helped in reassuring the authenticity of the approach.

Our analysis not only reveals a stage specific prioritized list of pathways but also highlights the underlying complexity associated with melanoma progression and reveals the important components that govern the complexity. Since the generated data contains numerous events with a huge number of novel relationships, we discussed the detail results of only the top-most relevant events to validate the network rewiring analysis. However, the complete results are available as a publicly available SQL database (M3db, S7 Fig) for further analysis. Our analysis and approach was different from existing pathway centric approaches that utilize curated information from different sources but single platform [43, 44]. The analytical approach discussed here is independent of the previous melanoma findings and strictly depends upon microarray analysis of the melanoma datasets. The approach validates several known network components and hence highlights the need to investigate the novel genes, gene interaction and gene set predictions emerging out of this analysis. However, we believe that the information from several other sources like transcription factor or protein-protein interaction can definitely aid in refining the current results, which is one of our future objectives.

## Conclusion

The analysis performed in the study demonstrates that melanoma genes can modulate its activity by changing network connections independent of altered gene expression. We also observed that the rewired genes are more consistent in expression and rewiring, as compared to DE genes and may even be more significant than DC genes in certain pathways. Moreover, the predicted functional gene sets are also statistically reproducible across multiple datasets, which indicates the robustness of our approach. Global increase in complexity of defined sub-network properties and gene count with melanoma progression is a common feature observed in all the sections of our analysis. This enabled us to reason how anti-melanoma drug target efficacy decreases once the melanoma cells program itself for metastasis. We were also able to successfully identify the genes that allow signalling pathways to cross-talk in different stages of melanoma. The predicted hub genes that are targets of known anti-neoplastic compounds too, may be explored for its potential anti-melanoma activity too.

## Materials and Methods

### Data collection, processing and differential expression

Expression profile datasets along with additional information were retrieved from different data using R package GEOquery [45] and ArrayExpress [46]. Expression data *X*_*n*_ = {*x*_1_,⋯,*x*_*n*_} was converted into R objects and processed with intra-array quantile normalization using Limma R package [47]. For each microarray sample probe IDs were converted to its respective entrez gene ID using platform annotation information section of GEO [48] or ArrayExpress [49] with careful manual observations. Multiple probes for a single gene were combined using *collapseRows* function of WGCNA R package, using default parameters [50]. We merged the datasets for further cross-platform microarray analysis using the common space across all the microarray datasets from diverse platforms. This was followed by inter-array intensity normalization and log transformation to obtain expression values in 16 bit resolution [51]. Since the values represent diverse microarray experiments and platforms, therefore we estimated the proportion of variance due to different experimental condition using PVCA R package [52]. The estimated batch effects were removed by rescaling the intensity values using C*ombat* function in SVA package [53]. Finally, we were able to generate a large dataset $\text{D}={\left\{x_{i},s_{i}\right\}}_{i=1}^{n}$ with *x*_*i*_ ∈^*N*^ the expression over N genes and *s*_*i*_ is the disease stage from meta-data available for each sample in respective literature. Dataset was stratified into four classes, *s* ∈ {N, CnM, CM, LN} with *p*_*1*_, *p*_*2*_, *p*_*3*_, *p*_*4*_ samples in each stage and progress as {N → CnM → CM → LN}. In order to remove the outlier sample(s), we performed standardized sample connectivities (*Z*.*K*) analysis of each sample, in each melanoma stage, which involves clustering of samples on the basis of biologically meaningful relationships defined by correlation matrix. The samples with *Z*.*K* values significantly lower (< -2.5) than other samples were removed from the analysis [54]. The processed data is available at http://dx.doi.org/10.6084/m9.figshare.1577546. The final *N × p* expression matrices were subjected to identify genes with a statistically different expression than normal using *lmfit* function of Limma package [55].

### Weighted gene co-expression network

Co-expression network or Graph (*G*) was mathematically represented as *G* = (*V*,*E*), where *V* is the set of nodes (i.e. genes) and *E* represents the numbers of nodes co-expressed or connected. The connectivity structure of graph was represented as adjacency matrix A*(G)*, where *A*_*ij*_ = *r*_*ij*_ if a node *i* and *j* are correlated with Pearson correlation coefficient (PCC; *r*) and *A*_*ij*_ = 0 otherwise. After removing self-loops, i.e. *A*_*ii*_ = 0, we performed the “tests of no correlation” for each PCC which tests the null hypothesis of *r*_*ij*_ = 0 and evaluated the subsequent p-value in the large sample approximation (*p* > 10), adjusted with Benjamini-Hochberg False Discovery Rate. Finally *r*_ij_> 0.5 and FDR adj.p < 0.05 were selected as a final edge between two connected nodes. Subsequent analysis on these weighted gene co-expression networks was performed using in-house R/PERL scripts and igraph (a R package) [56].

### Generation of random networks

In order to evaluate the significance of network measures, we generated random networks using Erdos-Renyi model with a same node degree distribution and node count as real network. In addition, the numbers of edges for each gene were also retained and only the connection types were scrambled. We defined elementary probability *p* = 〈*d*〉/*N* for connecting each node pair in each of the random graph, where N is the number of nodes and 〈*d*〉 is the average degree of real network. The significant differences between the degree distribution of real and random graphs were accessed by Kolmogorov-Smirnov test. Moreover, in order to evaluate if our networks follow power law distribution we subjected our networks for bootstrapping hypothesis test, using a R package poweRlaw [28].

### Calculation of differential connectivity using a random weighted gene co-expression network

For each node *i*, we measured its total connectivity (*K*) or degree with *j*-th nodes in each complex network, which represents the number of gene co-expressed with gene *i*. The connectivity can be measured in terms of adjacency matrix as:

$$K_{i}=\sum_{j=1}^{m}\left(A_{ij}\right)$$

The above step resulted into data vector $\left\{K_{i=1}^{n}\right\}$ representing the number of dependencies/degree for each node in networks. We measured the differential connectivity of a gene across two network conditions by the following definition:

$$dK_{i}{=K}_{i}^{d}-\;K_{i}^{c}$$

Where *K* ^d^ measures the connectivity in disease sample whereas K^c^ measures the connectivity in control network. We measured the significance of Differential Connectivity (DC) by calculating *dK*_*i*_ from random networks, generated by randomly assigning expression values from one group to another group and evaluating ${dK}_{i}^{\#}$ for each permutation. The above step was repeated 1000 times (*t* = 1000), sufficient to ensure random distribution of values. The statistical significance was evaluated by calculating the p-value using:

$$P_{i}=\;\Delta \left\{\left|{dK}_{i}^{\#}\right|\;>\;|{dK}_{i}\right|\}/t$$

Where Δ{S} represent the cardinality of set *S*. Each p-value was corrected for multiple hypothesis corrections using Benjamini-Hochberg False Discovery Rate (FDR) adjustment (adj.p value). Finally only the DC genes with adj.p < 0.01 were selected for further analysis.

#### Multi-edge rewiring

We executed overlapping of each gene and its connections in a disease state onto control network and evaluated the connection types. For each gene three different types of connections were obtained:

- Edges unique in disease state (EG).
- Edges lost by a gene in disease state (EL).
- Edges commonly displayed by both state.

For the statistical significant assessment of multi-edge gene circuitry rewiring, we selected genes ζ = *γ*_1_,⋯,*γ*_*g*_ with DC adj.p ≤ 0.05 and defined unique-edge count threshold for shortlisted genes ζ. The threshold used to define rewiring potential of a gene was calculated using power law distribution, which was approximated to novel connectivity of 70. Thus, the DC genes that gained more than 70 new edges were considered as rewired.

### Gene enrichment and sub-network analysis

The final selected rewired genes were allowed to mix with significantly up-regulated genes (logFC > 1 and adj.p < 0.05) whereas significantly down-regulated genes (logFC < -1 and adj.p < 0.05) were removed from the final gene lists of each cancer stage ζ^*s*^ = *γ*_1_,⋯,*γ*_*k*_. Each of the three gene lists ζ^*s*^ were subjected to a multi-way enrichment analysis using TargetMine webserver (build 20141221) [57] which utilizes three different data sources for pathway enrichment viz. KEGG [58], REACTOME [59], NCI-PID [60]. The initial analysis involves the identification of significantly enriched IPCs *I* ^*s*^ = *I*_1_, …, *I*_*T*_ (adj.p < 0.05), which represents the interconnected pathways sharing a common biological theme. The gene symbol IDs for each *I*_*t*_ were converted to its corresponding entrez gene IDs, which were mapped onto respective networks for module density and global transitivity analysis.

Each IPC is then split into its pathway component by re-submitting a gene list of each *I*_*t*_ in TargetMine and downloading an enriched pathway list. The entrez gene IDs for each pathway in a list for all the melanoma stages was mapped onto its respective stage network and normal network for calculating sub-network properties in both disease and normal network. We also connected two pathways if statistically significant number of genes is shared in the pathways, estimated by fisher’s exact test (p < 0.05).

Various fundamental network properties for each pathway were normalized in a single *R-*score to measure its overall relevance. *R-*score was defined as:

$$R=\text{log}(PC)+\frac{\emptyset_{d}-\emptyset_{c}}{\emptyset_{d}}+\;\frac{\partial_{d}-\partial_{c}}{\partial_{d}}$$

Where *PC* is the number of connected pathways, Ø is the per gene connectivity in disease (*d*) stage and control (*c*) stage, ∂ is the sub-network density of the connected nodes. For each melanoma stage, output of the calculations was a set of vector ${\left\{P_{t},R_{t}\right\}}_{t=1}^{T}$ where *R*_*t*_ corresponds to relevance of pathway *P*_*t*_ over all other *T-1*predicted pathways.

### Druggable genome analysis of hub genes

The most connected hub genes were selected from each of the predicted pathways and subjected for druggable genome analysis. We utilized cancerResource [39] and DGIdb [38] for mining the druggable genome (only cancer interactions) using our hub gene list for each metastatic melanoma stage. The final results generated by the aforementioned/above mentioned webservers were compiled into “gene-to-drug” and “drug-to-gene” lists, followed by manual screening for priori evidences for cancer relevant activity of the filtered genes and compounds known to target the genes.

## Supporting Information

S1 Fig Batch effect analysis. **A.** The proportion of variance in the dataset was analyzed before and after batch effect removal using top 10 principal components. **B.** PVCA estimation bar-chart analysis results suggesting the role of batches in explaining the dataset variation before batch adjustment. The graph reveals significant loss of such dataset variation after ComBat batch adjustment. **C.** The stage wise sample clustering (belonging to different platforms), before and after batch adjustment. Results suggest that experiments from different platforms overlap after batch adjustment. The different colors in each graph indicate different batches.
(TIFF)

S2 Fig Volcano plot representing the differential expression of CM stage as compared to CnM stage (logFC > 1 and adj.p value < 0.05). Significantly DE genes are represented as dark blue dots. CnM to CM transition involves sudden down-regulation of large number of genes and comparatively few genes show significant stage specific up-regulation including cancer biomarkers like *GDF15*.
(TIFF)

S3 Fig Gene Ontology (GO) analysis of DE genes. Left panel shows GO terms enriched (p value < 0.01) along with its gene count for up-regulated genes whereas right panel shows GO terms enriched for down-regulated genes. With melanoma progression, genes associated with skin development are down-regulated whereas genes related to cell adhesion, cell cycle and immune system are up-regulate.
(TIFF)

S4 Fig A. Distribution of Pearson Correlation Coefficient values (*r*) in different stage networks. Bottom graph shows comparison of correlation distribution density in all the four networks. B. Benjamini-Hochberg False Discovery Rate (FDR) as function of p-value in different stages of melanoma. (TIFF)

S5 Fig The numbers of unconnected network genes were identified by increasing the network threshold value in reference network. The network initiate to become sparse after *r*> = 0.4 and largest threshold that retain most of the connected components ranges from *r*> = 0.45–0.5.
(TIFF)

S6 Fig A. Comparison of degree distribution of four stage specific real networks with random networks (dotted lines; see materials and methods). CDF stands for Cumulative Distribution Function for the degree of connected genes in real and random networks. B. Scatter plot of degree and gene count for each network, which shows heavy tail distribution and only few genes are highly connected and hence these are hub genes, which resembles the characteristic of biological networks. P value represents the score to determine if a power law distribution is plausible for fitting heavy tailed distributions for a network. (TIFF)

S7 Fig M3db is a free web based platform for the analysis, visualization and retrieval of predicted dysregulated pathways in two exclusive metastatic melanoma stages (CM and LN). Processed or raw data at the different levels of the whole study can also be obtained in standard formats.
(TIFF)

S1 File Complete dataset details for all the stages of melanoma. (XLS)

S2 File List of differentially expressed genes in different melanoma stages. (XLS)

S3 File Integrated Pathway clusters predicted in different melanoma stages. (XLS)

S4 File Signalling Pathway connectivity for predicted dysregulated pathways in metastatic stages. (XLS)
