# Supplementary figures and images for: Gene Network Rewiring to Study Melanoma Stage Progression and Elements Essential for Driving Melanoma
Source: PLoS One. 2015 Nov 11;10(11):e0142443. doi: 10.1371/journal.pone.0142443 (PMC4641706; doi:10.1371/journal.pone.0142443)

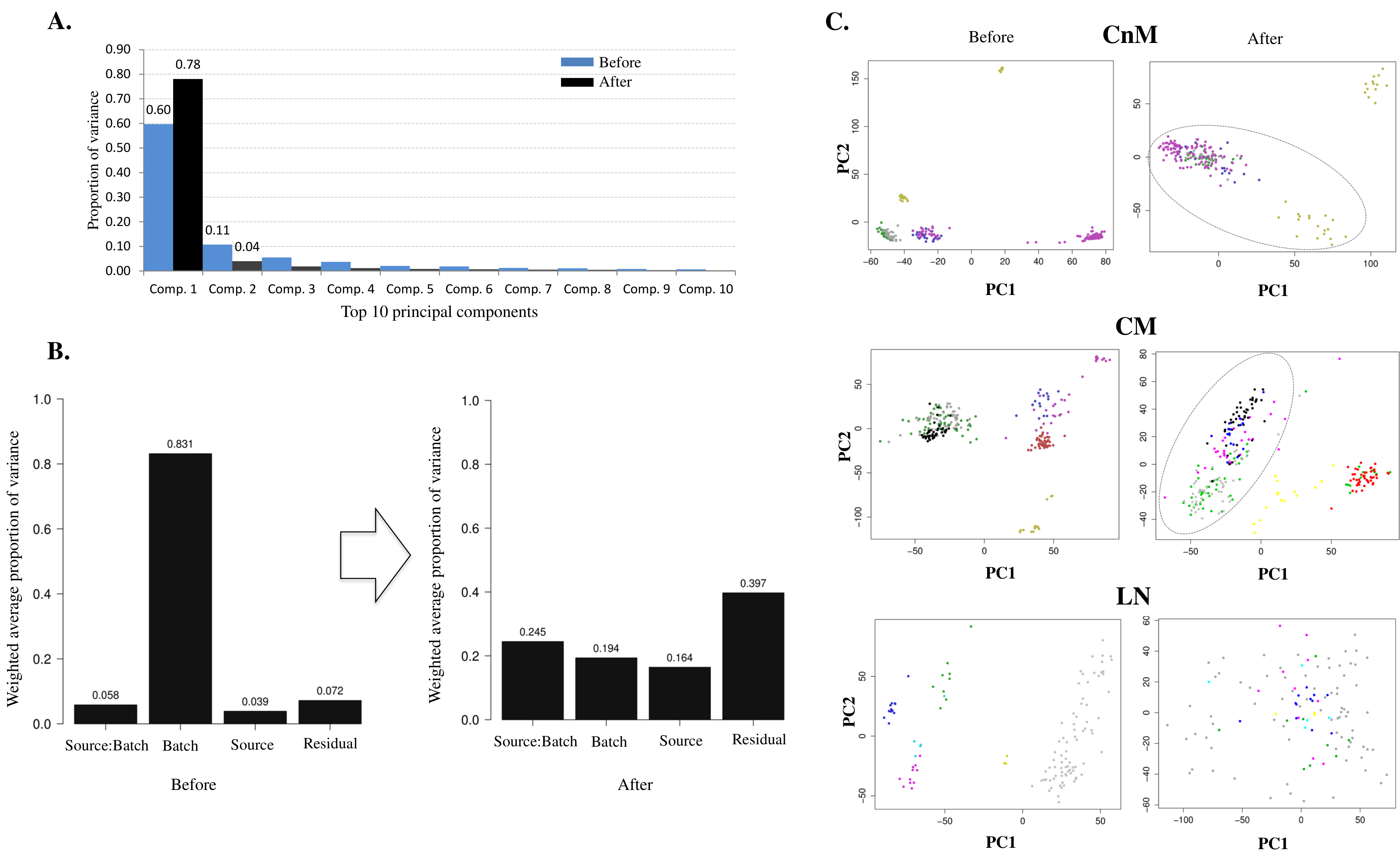

Supplement: S1 Fig — A. The proportion of variance in the dataset was analyzed before and after batch effect removal using top 10 principal components. B. PVCA estimation bar-chart analysis results suggesting the role of batches in explaining the dataset variation before batch adjustment. The graph reveals significant loss of such dataset variation after ComBat batch adjustment. C. The stage wise sample clustering (belonging to different platforms), before and after batch adjustment. Results suggest that experiments from different platforms overlap after batch adjustment. The different colors in each graph indicate different batches. (TIFF) [file pone.0142443.s001.tiff]

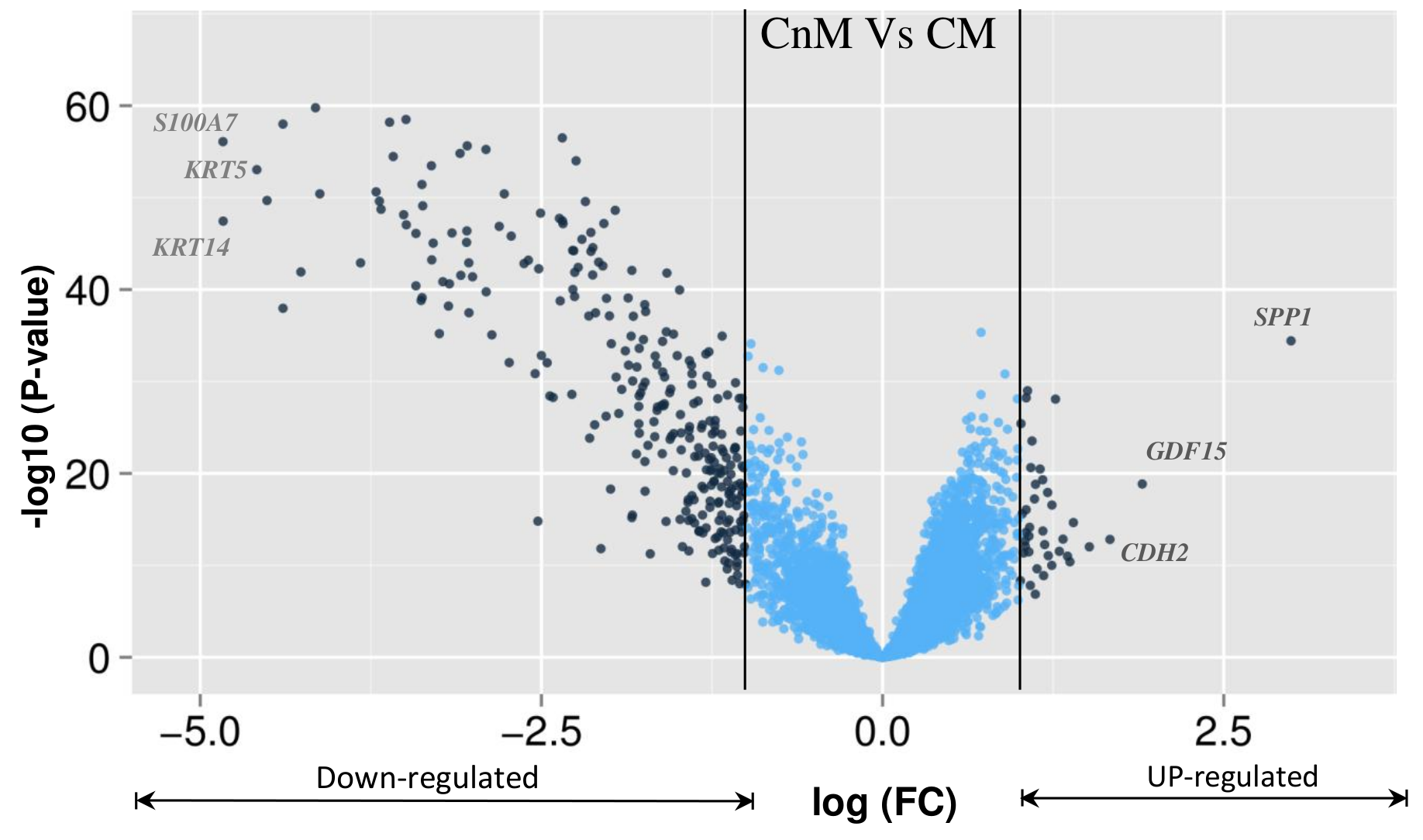

Supplement: S2 Fig — Significantly DE genes are represented as dark blue dots. CnM to CM transition involves sudden down-regulation of large number of genes and comparatively few genes show significant stage specific up-regulation including cancer biomarkers like GDF15. (TIFF) [file pone.0142443.s002.tiff]

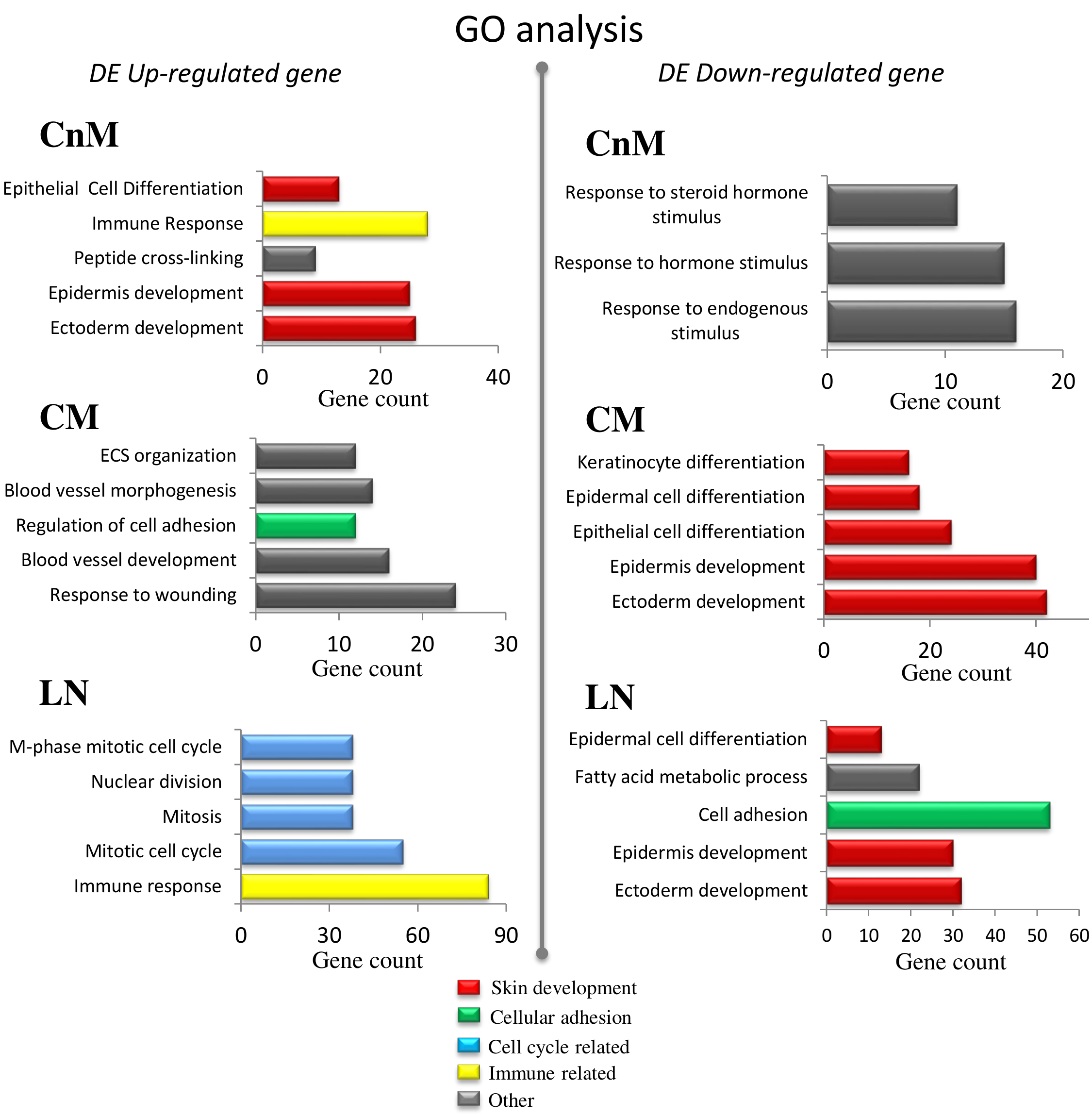

Supplement: S3 Fig — Left panel shows GO terms enriched (p value < 0.01) along with its gene count for up-regulated genes whereas right panel shows GO terms enriched for down-regulated genes. With melanoma progression, genes associated with skin development are down-regulated whereas genes related to cell adhesion, cell cycle and immune system are up-regulate. (TIFF) [file pone.0142443.s003.tiff]

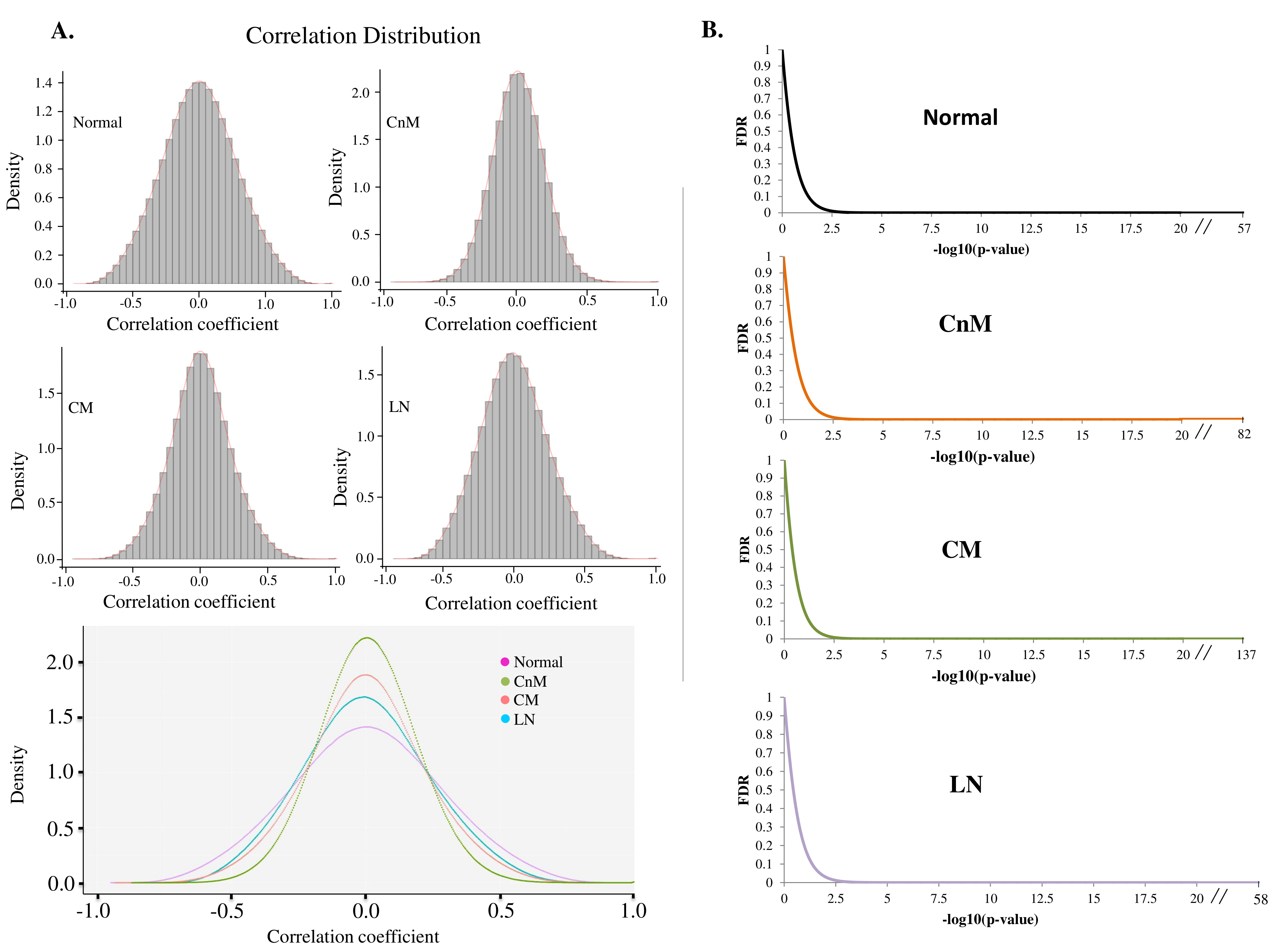

Supplement: S4 Fig — (TIFF) [file pone.0142443.s004.tiff]

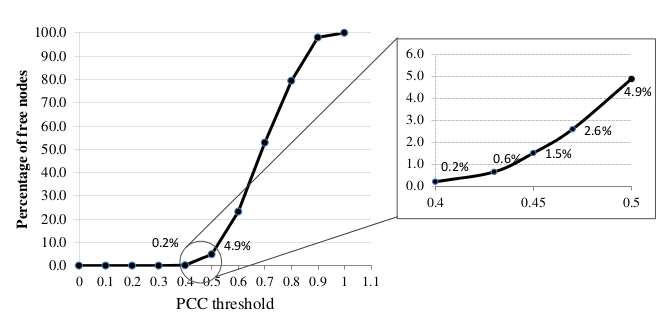

Supplement: S5 Fig — The network initiate to become sparse after r> = 0.4 and largest threshold that retain most of the connected components ranges from r> = 0.45–0.5. (TIFF) [file pone.0142443.s005.tiff]

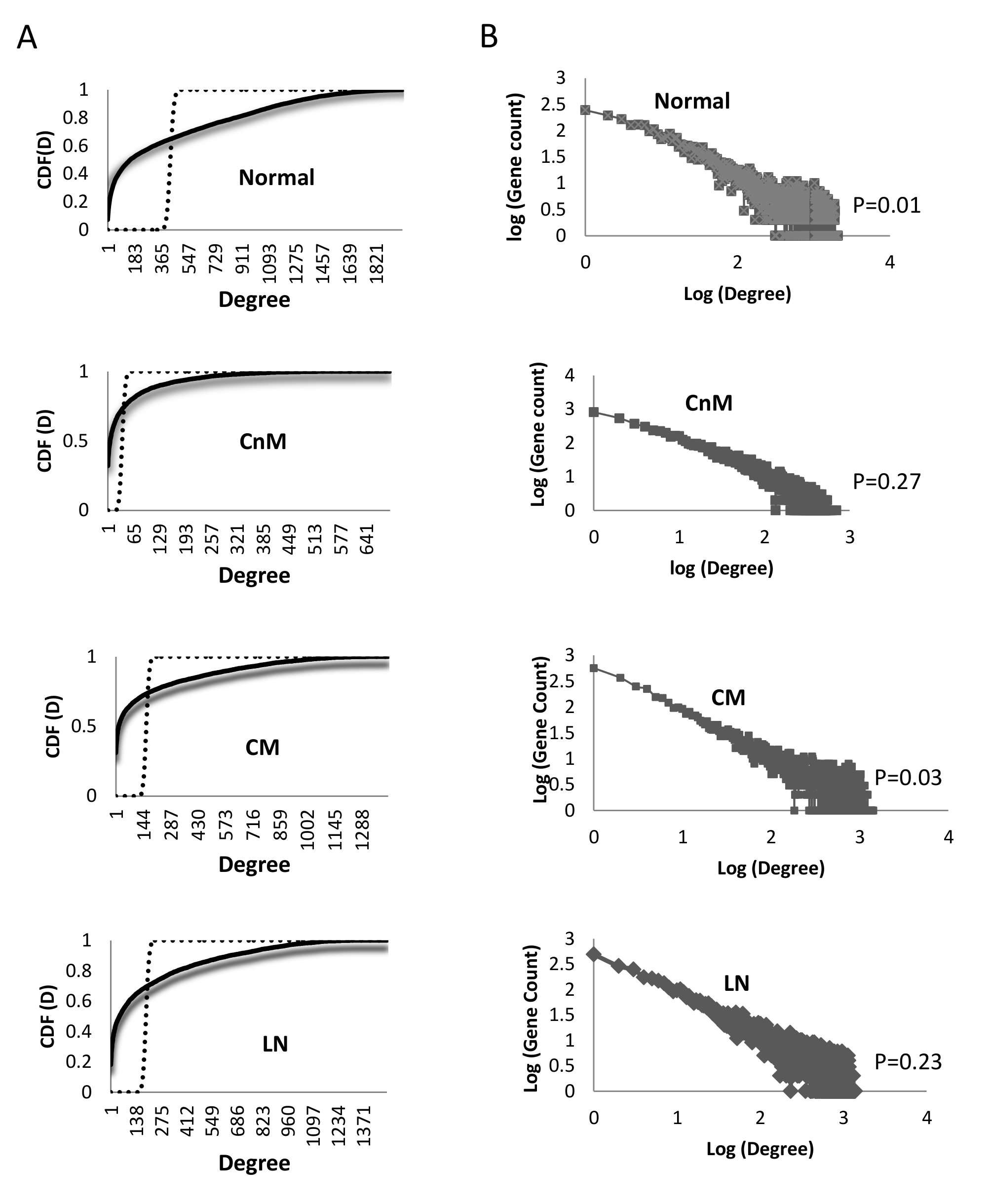

Supplement: S6 Fig — (TIFF) [file pone.0142443.s006.Tiff]

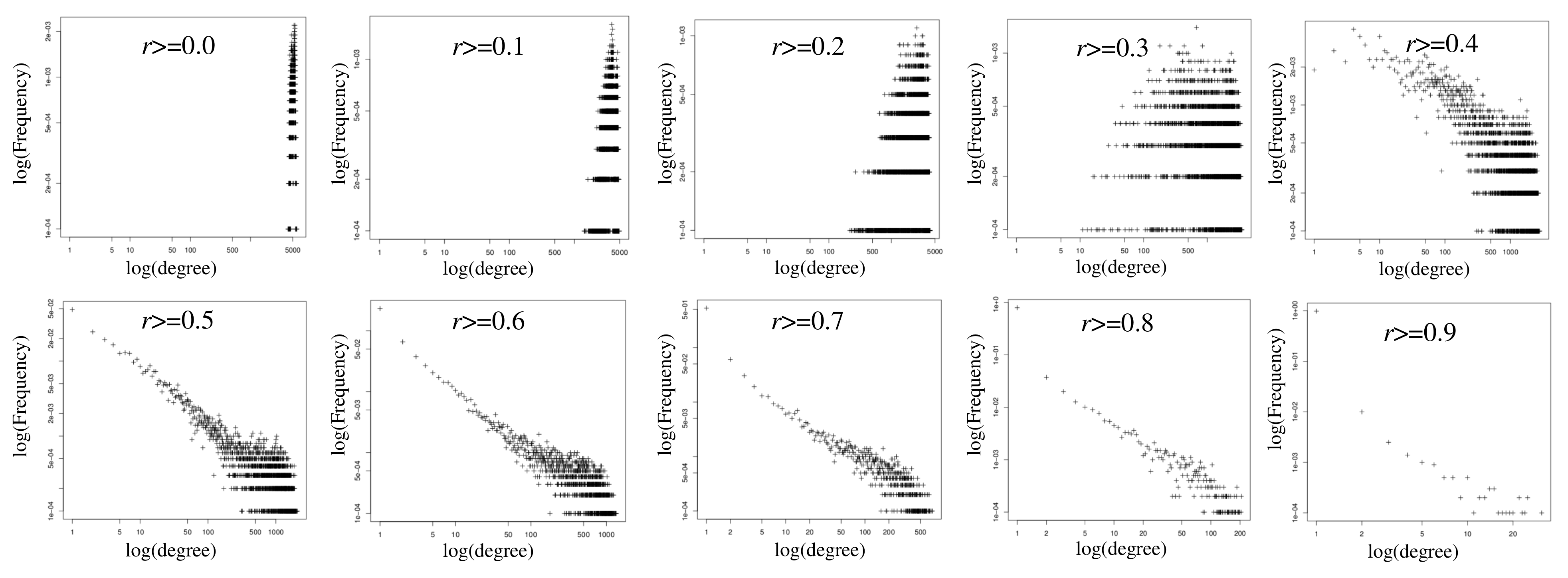

Supplement: S7 Fig — Processed or raw data at the different levels of the whole study can also be obtained in standard formats. (TIFF) [file pone.0142443.s007.tiff]
